# Mapping genomic regions affecting milk traits in Sarda sheep by using the OvineSNP50 Beadchip and principal components to perform combined linkage and linkage disequilibrium analysis

**DOI:** 10.1186/s12711-019-0508-0

**Published:** 2019-11-19

**Authors:** Mario Graziano Usai, Sara Casu, Tiziana Sechi, Sotero L. Salaris, Sabrina Miari, Stefania Sechi, Patrizia Carta, Antonello Carta

**Affiliations:** Genetics and Biotechnology-Agris Sardegna, Loc. Bonassai S.S. 291 Sassari-Fertilia – Km. 18.600, 07100 Sassari, Italy

## Abstract

**Background:**

The detection of regions that affect quantitative traits (QTL), to implement selection assisted by molecular information, remains of particular interest in dairy sheep for which genetic gain is constrained by the high costs of large-scale phenotype and pedigree recording. QTL detection based on the combination of linkage disequilibrium and linkage analysis (LDLA) is the most suitable approach in family-structured populations. The main issue in performing LDLA mapping is the handling of the identity-by-descent (IBD) probability matrix. Here, we propose the use of principal component analysis (PCA) to perform LDLA mapping for milk traits in Sarda dairy sheep.

**Methods:**

A resource population of 3731 ewes belonging to 161 sire families and genotyped with the OvineSNP50 Beadchip was used to map genomic regions that affect five milk traits. The paternally and maternally inherited gametes of genotyped individuals were reconstructed and IBD probabilities between them were defined both at each SNP position and at the genome level. A QTL detection model fitting fixed effects of principal components that summarize IBD probabilities was tested at each SNP position. Genome-wide (GW) significance thresholds were determined by within-trait permutations.

**Results:**

PCA resulted in substantial dimensionality reduction, in fact 137 and 32 (on average) principal components were able to capture 99% of the IBD variation at the locus and genome levels, respectively. Overall, 2563 positions exceeded the 0.05 GW significance threshold for at least one trait, which clustered into 75 QTL regions most of which affected more than one trait. The strongest signal was obtained for protein content on *Ovis aries* (OAR) chromosome 6 and overlapped with the region that harbours the casein gene cluster. Additional interesting positions were identified on OAR4 for fat content and on OAR11 for the three yield traits.

**Conclusions:**

PCA is a good strategy to summarize IBD probabilities. A large number of regions associated to milk traits were identified. The outputs provided by the proposed method are useful for the selection of candidate genes, which need to be further investigated to identify causative mutations or markers in strong LD with them for application in selection programs assisted by molecular information.

## Background

The identification of genomic regions that affect traits of interest and the application of marker- or gene-assisted selection [1] in livestock are crucial to speed up genetic improvement. This is especially valid in species for which genetic gain is hampered by the relatively high costs of large-scale phenotyping and the logistic constraints of artificial insemination [2]. However, application of selection assisted by molecular information for traits that are influenced by numerous loci, each one explaining a small portion of the trait variance, is limited by the lack of power of experiments based on low-density marker maps [3]. In sheep, attempts to identify regions that affect quantitative traits (QTL) were performed first by using microsatellite maps [4–13]. Most of the identified QTL showed low significance levels and large confidence intervals, and thus their use in selection programs was not possible [2].

Nevertheless, the discovery of thousands of single-nucleotide markers (SNPs) and cost-effective tools (SNP arrays) to genotype them on a large number of animals as well as the recent availability of affordable whole-genome sequencing techniques, has opened new perspectives. It is expected that polymorphisms with small effects that collectively explain an increasing amount of the genetic variance may be gradually identified by using denser molecular marker maps on larger structured resource populations [14]. Thus, individuals that belong to pre-existing and new dairy sheep experimental populations have been accurately recorded for several traits and genotyped with 50 K and/or 600 K SNP chips. This is the case of an experimental flock of Sarda ewes, which has been set up since 2000 as a resource population to implement selection assisted by molecular information in the breeding scheme of this Italian dairy breed. QTL detection studies based on SNP arrays and the availability of increasingly accurate genome asec15 nnotation data allow the listing of potential candidate genes [15–20]. Moreover, whole-genome re-sequencing of target animals has been used to restrict the number of candidate polymorphisms. Thus, putative causative mutations that affect traits of economic interest have been proposed [21–24].

Among the available QTL detection approaches, those based on the combined use of linkage disequilibrium (LD) and linkage analysis (LA) information (LDLA) have been indicated as the most powerful, robust and precise in populations that are structured in families [16, 25, 26]. The main reason is that they account for both recombination events that occurred within genotyped generations and historical recombination events that occurred in generations prior to genotyping [25]. Several approaches have been proposed to combine LD and LA information [25, 27–31]. The classical LDLA method [25, 32] performs a variance component analysis at each putative QTL position by using identity-by-descent (IBD) probabilities between haplotypes. The main issue is that the IBD probability matrix is often dense, non-positive definite and computationally demanding for its inversion [29, 33]. Thus, either strategies that perform the hierarchical clustering of haplotypes based on IBD probability [33–35] or the approximation of the IBD based on the extent of the identity-by-state status between haplotypes [36] have been used. However, these approximations inevitably result in a loss of information [29].

An alternative way to process IBD information is principal component analysis (PCA), which has the desirable feature of collapsing information that is contained in a set of correlated variables by a smaller set of orthogonal variables. As such, PCA has been proposed as a technique to reduce the dimensionality of predictors in genomic selection [37, 38].

The aim of this study was to detect genomic regions that affect milk traits in the resource population of Sarda sheep by applying an LDLA approach combined with PCA to overcome computational issues of the IBD matrix and minimize the loss of information.

## Methods

### Resource population

The generation of the resource population (RP) started in 1999 when 10 Lacaune × Sarda F1 sires were mated to Sarda ewes to produce 928 back-cross female lambs in the framework of an European project aimed at detecting QTL in the main European sheep breeds (QLK5-CT-2000-00656; “genesheepsafety”). Subsequently, we focused on the detection of QTL segregating in the pure Sarda breed, and since 2002 we used exclusively Sarda rams (SA) to produce the yearly replacement of RP. Until 2009, the average size of the sire families was 43 daughters whereas, from 2010 onward the average size of families decreased to nine daughters, in order to increase the number of Sarda *bloodlines* represented in the RP. Sarda sires were always chosen based on their genetic impact on the registered population among rams belonging to the artificial insemination centre of the breed.

In total, 3949 ewes from 161 rams (10 F1 and 151 SA) were generated until 2015. Ewes of RP were kept until the 4th (occasionally the 5th) lactation on an experimental farm. The farming system was similar to that commonly applied in Sardinia with most of the adult ewes lambing in autumn and yearlings lambing between January and March. The ewes were milked twice a day by machine from weaning (3–4 weeks from lambing) until the end of July. The feeding regime was based on controlled grazing, supplemented by hay and concentrates in winter and late spring.

### Genotypes and phenotypes

All the ewes of RP and their sires as well as the 10 Lacaune sires of F1 and 11 Sarda sires of SA were genotyped with the Illumina Inc. OvineSNP50 Beadchip. SNP editing was performed using call rate and MAF thresholds of 95% and 1%, respectively. The ovine genome assembly v4.0 and the software SNPchimMpv.3 [39] were used to construct the genetic map by assuming 1 Mb = 1 cM. Unmapped SNPs and SNPs on sex chromosomes were not included in the study.

A large range of traits of economic interest was measured in the RP. In the current study, we focused on milk traits: milk yield (MY); fat yield (FY); protein yield (PY); fat content (FP) and protein content (PP). MY, FP and PP were measured twice a month during the milking period at the am and pm milking. Lactation records were computed by the Fleischmann method, using records from the milking period only (in agreement with ICAR recommendations), by considering an initial suckling period of 30 days. Finally, 13,059 lactations of 3731 ewes recorded from 2000 to 2017 were retained. The average number of records per ewe was 3.5 ± 1.02, ranging from 1 (5% of animals) to 5 (9.3% of animals); most of the ewes (55.5%) had four records.

First, in order to adjust for the main environmental effects, raw lactation records were analysed with single-trait repeatability animal models using the ASReml 4.1 software [40]. Genetic relationships between animals were taken into account by calculating the genomic relationship matrix [41] between 4513 animals, including F1 and SA sires and their genotyped ancestors. The animal model included as fixed effects the year-management-group interaction (37 levels), the year-month of lambing-parity-age class interaction (230 levels) and the milking length within age class (adult and primiparous) as a covariate. The average performance deviation (APD) of each ewe was calculated as the average of lactation records adjusted for fixed effects. The APD used in this study as pseudo-phenotypes for QTL detection differ from the yield deviations [42] that were used in similar studies in that the performances are not adjusted for permanent environmental effects in order to prevent inaccurate estimations due to the likely confounding between permanent environment and additive genetic effects. Indeed, Pearson’s correlations between additive genetic and permanent environmental effects from the repeatability animal model ranged, for the five analyzed traits, from 0.46 for FY to 0.50 for PP. Moreover, although in this study we shall investigate only additive effects, ADP include dominance and epistatic genetic effects when they exist and are suitable pseudo-phenotypes for testing such effects in further analyses. Finally, 3731 APD from as many ewes were available. To verify the suitability of the applied animal model, the ratio between the estimated genomic and total variance was compared with the heritabilities reported in the literature. In the same way, the correlations between APD and GEBV of different traits were compared with phenotypic and genetic correlations estimated in other studies.

### Classification of gametes and reconstruction of gametic phases

By “gamete”, we refer to the whole haploid set of autosomes that are inherited by an individual from one of the two parents. Moreover, we classified the gametes of the population as base haplotypes (BH) when inherited from an ungenotyped parent and replicated haplotypes (RH) when inherited from a genotyped parent. The pool of BH comprised both gametes of F1 rams and of 63 SA rams, the maternal or paternal gametes of the 35 ewes with an unknown sire or dam, respectively, and the maternal gametes of the 928 back-cross ewes and of 85 SA rams for which the sire was genotyped. Finally, 1207 gametes were classified as BH, i.e. the 10 F1 sires paternal Lacaune gametes (${\text{BH}}^{\text{L}}$) and 1197 Sarda origin gametes (${\text{BH}}^{\text{S}}$). Then, all 7462 gametes (${\text{n}}_{\text{RH}}$) carried by the 3731 ewes with production records (${\text{n}}_{\text{P}}$) were considered as replicates (RH) of the 1207 BH (${\text{n}}_{\text{BH}}$). An example of how gametes were classified is given in Appendix.

The paternal and maternal inherited gametes of all the genotyped individuals were reconstructed by using a procedure based on the linkage disequilibrium multilocus iterative peeling method proposed by Meuwissen and Goddard [43]. In this method, the parental origin of the alleles carried by an individual is iteratively inferred on the genotypes of parents and offspring at a given locus if they are already phased or at the neighbouring phased loci if the phase at the given locus is unknown. Here, the LD at the population level was ignored, since the population structure was expected to allow a high level of precision using family relationships only. For individuals with both parents without a genotype, the paternal or maternal origin was arbitrary assigned. Genotypes for which the parental origin of alleles was assigned with a probability lower than 0.99 were assumed missing.

### Calculation of IBD probabilities

The marked familial structure of the RP led us to exploit the information from the within-family linkage analysis (LA) in addition to that from the population-wide linkage disequilibrium (LD) to estimate IBD probabilities.

First, IBD probabilities between BH and RH were calculated by LA (${\text{IBD}}_{\text{LA}}$) given the known gametic phases and the pedigree information. The grand-parental origin of each RH was estimated at each SNP position with certainty when the genotype at a given position was not missing and the parent transmitting RH was heterozygous. When these conditions were not fulfilled, the probability of a grand-parental origin at a given locus was determined based on information from the closest neighbouring informative loci [44, 45]. Then, transmission from BH to RH was traced through generations following Fernando and Grossman [46]. At each SNP position $l$, ${\text{IBD}}_{\text{LA}}$ probabilities were stored in a matrix ${\mathbf{H}}_{l}$ with size ${\text{n}}_{\text{RH}} \times {\text{n}}_{\text{BH}}$. Moreover, the number of replicates of a given BH in RP at each SNP position $l$ (${\mathbf{f}}_{l}$) was calculated as ${\mathbf{f}}_{l} = {\mathbf{H}}_{l}^{\varvec{'}} 1$, where $\bf{1}$ is a vector of ${\text{n}}_{\text{RH}}$ ones (see Appendix).

Secondly, IBD between BH were estimated by LD analysis (${\text{IBD}}_{\text{LD}}$) at each SNP position following Meuwissen and Goddard [47]. The ${\text{IBD}}_{\text{LD}}$ probability was conditioned to the identity-by-state (IBS) status of neighbouring SNPs using windows of 21 SNPs (10 upstream and 10 downstream resulting in an average window length of 1 Mb) and to the within-breed (Lacaune or Sarda) expected homozygosity. The ${\text{IBD}}_{\text{LD}}$ between ${\text{BH}}^{\text{S}}$ and ${\text{BH}}^{\text{L}}$ were assumed to be null. At each SNP position $l$, ${\text{IBD}}_{\text{LD}}$ probabilities were stored in a matrix ${\mathbf{U}}_{l}$ with size ${\text{n}}_{\text{BH}} \times {\text{n}}_{\text{BH}}$.

Once we had precisely estimated the covariances between BH and between BH and RH as well as the BH number of replicates at each locus, the average values across loci were also calculated and stored in ${\mathbf{U}}_{g}$ (${\text{n}}_{\text{BH}} \times {\text{n}}_{\text{BH}}$), ${\mathbf{H}}_{g}$ (${\text{n}}_{\text{RH}} \times {\text{n}}_{\text{BH}}$) and ${\mathbf{f}}_{g}$ (${\text{n}}_{\text{BH}}$), respectively. These matrices will be used later to estimate genome-wide IBD probabilities between gametes to adjust for the polygenic effect of background genes.

### Principal component analysis and QTL detection model

Hereafter we describe a novel LDLA approach for QTL mapping that, similarly to the basic LDLA model proposed by Meuwissen et al. [25], relies on the IBD information at the locus level and takes the effect of background genes into account. In their study, Meuwissen et al. [25] modelled the phenotypic records by the random effects of all the inherited gametes ($\mathbf{h}$, twice the number of individuals) at a given position $l$ and the random polygenic effects ($\mathbf{u}$, i.e. the combined effect of background genes) of all the individuals. In the original model [25]: ${\mathbf{y}} = \bf {1} \mu+ {\mathbf{Zh}} + {\mathbf{u}} + {\mathbf{e}}$, the covariance matrix of gametic effects ${\mathbf{G}}_{l}$ included IBD probabilities between founder gametes that were obtained by LD analysis (${\text{IBD}}_{\text{LD}}$) [47] and IBD probabilities between founder and non-founder gametes and between non-founders gametes that were obtained by combining the corresponding transmission probabilities (${\text{IBD}}_{\text{LA}}$) with ${\text{IBD}}_{\text{LD}}$, using the algorithm described by Fernando and Grossman [46]. The additive polygenic covariance between individuals was considered through the numerator relationship matrix ${\mathbf{A}}$ based on pedigree information. In the basic method [25], the maximum likelihood estimates of the variance components were calculated at each putative QTL position $l$. The application of this method implies some relevant issues related to the nature of the ${\mathbf{G}}_{l}$ matrix, which is usually dense and may turn out to be non-positive definite, and the computational needs in applying the variance component analysis at each investigated position. To overcome these issues, we propose a novel approach which uses the principal component analysis (PCA) to handle ${\mathbf{G}}_{l}$ and exploits the dimensional reduction of the model equations that may be achieved by PCA to estimate both QTL and polygenic effects. First, PCA is used to capture the IBD information at the locus level with the aim of overcoming the difficulties in inverting the ${\mathbf{G}}_{l}$ matrix, in a different way from previous strategies having the same purpose [33–36, 48] which frequently result in a loss of information [29]. Second, PCA is applied to the matrix of the genome- wide IBD probabilities between gametes (i.e. the average across loci of IBD probabilities locus-wide) which is used instead of the classical numerator relationship matrix (${\mathbf{A}}$) to take into account the polygenic effects. A similar approach was used by Rothammer et al. [49, 50], which applied PCA to reduce the dimension of the relationship matrix and used explanatory PC as fixed effects in their QTL detection model. Third, PC that explain most of the variability of both the locus-level and genome-wide IBD probability matrices are included in the model as fixed effects to estimate both QTL and polygenic effects by performing a least square analysis instead of the more computationally demanding variance component one.

Thus, at each SNP position $l$ the model is the following:

1 $${\mathbf{y}} = \bf{1}\mu + {\mathbf{ZV}}_{l} {\varvec{\upbeta}}_{l} + {\mathbf{ZV}}_{g} {\varvec{\upalpha}}_{l} + {\varvec{\upvarepsilon}}$$

where ${\mathbf{y}}$ is a vector of APD of ${\text{n}}_{\text{p}}$ ewes for MY, PY, FY, PP and FP; $\mu$ is the overall mean; ${\varvec{\upbeta}}_{l}$ is a vector of the fixed effects of the ${\text{n}}_{{{\text{PC}}_{l} }}$ principal components that explain more than 99% of the within breed variation (${\text{PC}}_{l}$) of the IBD probability matrix ${\mathbf{G}}_{l}$, i.e. ${\varvec{\upbeta}}_{l}$ summarizes the effects of haplotypes at the QTL position $l$; ${\varvec{\upalpha}}_{l}$ is a vector of the fixed effects of the ${\text{n}}_{{{\text{PC}}_{g} }}$ principal components that explain more than 99% of the variation (${\text{PC}}_{g}$) of the genome-wide IBD probability matrix, i.e. ${\varvec{\upalpha}}_{l}$ summarizes the polygenic effects of the gametes; $\bf{1}$ is a vector of ${\text{n}}_{\text{p}}$ ones; ${\mathbf{Z}}$ is a ${\text{n}}_{\text{p}} \times {\text{n}}_{\text{RH}}$ incidence matrix relating phenotypes with RH; ${\mathbf{V}}_{l}$ is a ${\text{n}}_{\text{RH}} \times {\text{n}}_{{{\text{PC}}_{l} }}$ matrix including the ${\text{PC}}_{l}$ scores of RH, ${\mathbf{V}}_{g}$ is a ${\text{n}}_{\text{RH}} \times {\text{n}}_{{{\text{PC}}_{g} }}$ matrix including the ${\text{PC}}_{g}$ scores of RH; ${\varvec{\upvarepsilon}}$ is a vector of ${\text{n}}_{\text{p}}$ residuals assuming that ${\varvec{\upvarepsilon}}\sim {\text{N}}\left( {0,\sigma_{\upvarepsilon}^{2} {\mathbf{R}}^{ - 1} } \right)$ with ${\mathbf{R}}$ a diagonal matrix with the APD’s reliability ($r$) as diagonal element. For each investigated trait (MY, PY, FY, PP and FP), reliabilities were calculated as $r_{\text{i}} = 1 - {\text{se}}\left( {{\hat{\text{a}}}_{\text{i}} } \right)^{2} /\sigma_{\text{a}}^{2}$, from a repeatability linear model ${\text{y}}_{\text{ij}} = {\text{a}}_{\text{i}} + {\text{e}}_{\text{ij}}$, where ${\text{y}}_{\text{ij}}$ is the performance deviation ${\text{j}}$ (i.e. the lactation record ${\text{j}}$ adjusted for the fixed effects estimated with the full animal model) of ewe ${\text{i}}$, ${\text{a}}_{\text{i}}$ is the random ewe effect assuming that ${\mathbf{a}}\sim {\text{N}}\left( {0, \sigma_{\text{a}}^{2} {\mathbf{I}}} \right)$ and ${\text{e}}_{\text{ij}}$ is the corresponding error, assuming that ${\mathbf{e}}\sim {\text{N}}\left( {0, \sigma_{\text{e}}^{2} {\mathbf{I}}} \right)$.

Below, we explain how the PC scores of the ${\mathbf{V}}_{l}$ and ${\mathbf{V}}_{g}$ matrices were calculated. In addition, a numerical example is given in Appendix.

As far as the ${\mathbf{V}}_{l}$ elements are concerned, in order to limit the computational requirements to extract PC directly from the large (${\text{n}}_{\text{RH}} \times {\text{n}}_{\text{RH}}$) ${\mathbf{G}}_{l}$ matrix, the PCA was performed on a ${\text{n}}_{\text{BH}} \times {\text{n}}_{\text{BH}}$ matrix denoted as ${\mathbf{U}}_{l}^{w}$, where the ${\text{IBD}}_{\text{LD}}$ probabilities between BH, stored in ${\mathbf{U}}_{l}$, were weighted for the ${\text{IBD}}_{\text{LA}}$ probabilities between BH and RH by condensing ${\mathbf{H}}_{l}$ information in a ${\text{n}}_{\text{BH}} \times {\text{n}}_{\text{BH}}$ diagonal matrix ${\mathbf{F}}_{l}$, in which the diagonal elements are the number of RH of each BH (stored in ${\mathbf{f}}_{l}$, where ${\mathbf{f}}_{l} = {\mathbf{H}}_{l}^{\varvec{'}} 1$).

The ${\mathbf{U}}_{l}^{w}$ matrix is defined as:

2 $${\mathbf{U}}_{l}^{w} = {\mathbf{F}}_{l}^{1/2} {\mathbf{U}}_{l} {\mathbf{F}}_{l}^{1/2} .$$

PCA was carried out on ${\mathbf{U}}_{l}^{w}$ by using the Jacobi algorithm. Eigenvectors (${\mathbf{V}}_{l}^{w}$) relating to the largest principal components that together explain more than 99% of the within-breed variation (${\text{PC}}_{l}$) were retained. Finally, ${\text{IBD}}_{\text{LA}}$ probabilities between BH and RH (${\mathbf{H}}_{l}$) were combined with ${\mathbf{V}}_{l}^{w}$ to define the ${\text{PC}}_{l}$ scores of RH (${\mathbf{V}}_{l}$) by:

3 $${\mathbf{V}}_{l} = {\mathbf{H}}_{l} {\mathbf{F}}_{l}^{ - 1/2} {\mathbf{V}}_{l}^{w} .$$

Note that when ${\text{IBD}}_{\text{LA}}$ between BH and RH are estimated with certainty and, thus, ${\mathbf{H}}_{l}$ only contains 0 and 1, then: ${\mathbf{H}}_{l} {\mathbf{U}}_{l} {\mathbf{H}}_{l}^{'} = {\mathbf{G}}_{l}$ and ${\mathbf{F}}_{l} = {\mathbf{H}}_{l}^{'} {\mathbf{H}}_{l}$; eigenvalues from ${\mathbf{G}}_{l}$ correspond to eigenvalues from ${\mathbf{U}}_{l}^{w}$ for the explanatory principal components (${\text{PC}}_{l}$) and ${\text{PC}}_{l}$ scores from ${\mathbf{G}}_{l}$ correspond to ${\mathbf{V}}_{l}$ (see Appendix). When ${\text{IBD}}_{\text{LA}}$ between BH and RH are estimated without certainty and ${\mathbf{H}}_{l}$ contains intermediate values between 0 and 1, ${\text{PC}}_{l}$ scores from ${\mathbf{G}}_{l}$ and ${\mathbf{V}}_{l}$ do not correspond perfectly and differences tend to increase as the uncertainty of ${\text{IBD}}_{\text{LA}}$ probabilities increases. This is because ${\mathbf{U}}_{l}^{w}$ only considers the covariance that derives from ${\text{IBD}}_{\text{LD}}$ excluding the covariance between RH pairs generated by imprecise estimation of transmissions of BH. This effect is negligible in our experiment because most transmission probabilities are estimated with certainty.

Since the ${\text{IBD}}_{\text{LD}}$ between ${\text{BH}}^{\text{S}}$ and ${\text{BH}}^{\text{L}}$ was set to 0 and two sets of breed-specific PC were obtained, the matrix ${\mathbf{V}}_{l}$ can be detailed as $\left[ {{\mathbf{V}}_{l}^{\text{S}} {\mathbf{V}}_{l}^{\text{L}} } \right]$. where ${\mathbf{V}}_{l}^{\text{S}}$ and ${\mathbf{V}}_{l}^{\text{L}}$ are the ${\text{PC}}_{l}$ summarising IBD probabilities between the gametes of Sarda and Lacaune origin, respectively. In Eq. (1) ${\mathbf{V}}_{l}$ elements, which are related by the incidence matrix ${\mathbf{Z}}$ to phenotypes, are used as covariates on the investigated traits to estimate QTL effects at locus $l$ (${\varvec{\upbeta}}_{l}$).

As far as the ${\mathbf{V}}_{g}$ elements are concerned, the PCA performed directly on the weighted genome-wide ${\text{IBD}}_{\text{LD}}$ matrix, (${\mathbf{U}}_{g}^{w}$) computed as in Eq. (2) resulted in 1022 PC that were needed to capture 99% of the total variation. This limited dimensional reduction is due to the moderate genome-wide ${\text{IBD}}_{\text{LD}}$. probabilities between BH (on average around 0.1) and the small number of replicates of some BH on RP. Thus, in order to not over-parameterize the model, we considered the BH with the highest impact on RP (${\text{BH}}_{\text{h}}$). Then, to recover information from BH with few RH, a matrix of coefficients ${\mathbf{W}}$ relating ${\text{BH}}_{\text{h}}$ to all the BH was calculated as:

4 $${\mathbf{W}} = {\mathbf{U}}_{{g\__{h} }} {\mathbf{U}}_{{g\__{hh} }}^{ - 1} ,$$

where ${\mathbf{U}}_{g\_h}$ is the section of ${\mathbf{U}}_{g}$ including ${\text{IBD}}_{\text{LD}}$ probabilities between all the BH with ${\text{BH}}_{\text{h}}$ and ${\mathbf{U}}_{g\_hh}^{ - 1}$ is the inverse of the section of ${\mathbf{U}}_{g}$ including ${\text{IBD}}_{\text{LD}}$ probabilities between ${\text{BH}}_{\text{h}}$ pairs. The average number of replicates per ${\text{BH}}_{\text{h}}$ was then updated as ${\mathbf{f}}_{g\_h} = {\mathbf{W^{\prime}f}}_{g}$. The ${\text{BH}}_{\text{h}}$ set was iteratively selected as the smallest group of BH that satisfied the condition that $\sum {\mathbf{f}}_{g\_h} /{\text{n}}_{\text{RH}}$ is higher than 0.99. According to the analysis at the locus level, the Jacobi algorithm was performed on the matrix ${\mathbf{U}}_{g\_hh}^{w}$ computed as:

5 $${\mathbf{U}}_{g\_hh}^{w} = {\mathbf{F}}_{g\_h}^{1/2} {\mathbf{U}}_{{g\_hh\varvec{ }}} {\mathbf{F}}_{g\_h}^{1/2} ,$$

where ${\mathbf{F}}_{g\_h}$ is a diagonal matrix, with its diagonal elements being the number of replicates stored in ${\mathbf{f}}_{g\_h}$. Eigenvectors (${\mathbf{V}}_{g\_hh}^{w}$) of the largest principal components that together explain more than 99% of the total variation of ${\mathbf{U}}_{g\_hh}^{w}$ (${\text{PC}}_{g}$) were retained. Finally, genome-wide ${\text{IBD}}_{\text{LA}}$ probabilities between BH and RH (${\mathbf{H}}_{g}$) were combined with ${\mathbf{V}}_{g\_hh}^{w}$ to define ${\text{PC}}_{g}$ scores of RH (${\mathbf{V}}_{g}$) by:

6 $${\mathbf{V}}_{g} = {\mathbf{H}}_{g} {\mathbf{WF}}_{g\_h}^{ - 1/2} {\mathbf{V}}_{g\_hh}^{\text{w}} .$$

In Eq. (1), ${\mathbf{V}}_{g}$, scores which are related by the incidence matrix ${\mathbf{Z}}$ to phenotypes, are used as covariates on the investigated traits to estimate polygenic effects (${\varvec{\upalpha}}_{l}$).

Note that ${\varvec{\upbeta}}_{l}$ and ${\varvec{\upalpha}}_{l}$ vectors in Eq. (1) are both fixed effects that are depicted separately to highlight that the model aims at estimating QTL effects (${\varvec{\upbeta}}_{l}$) while adjusting for the background of polygenes (${\varvec{\upalpha}}_{l}$). Moreover, covariates related to ${\varvec{\upbeta}}_{l}$ $\left( {{\mathbf{ZV}}_{l} } \right)$ are locus-specific whereas covariates related to ${\varvec{\upalpha}}_{l}$ $\left( {\mathbf{ZV}_{g} } \right)$ remain constant throughout the genome.

In addition, in accordance with ${\mathbf{V}}_{l}$, ${\varvec{\upbeta}}_{l}^{'}$ can be detailed as $\left[ {{\varvec{\upbeta}}_{l}^{{'{\text{S}}}} {\varvec{\upbeta}}_{l}^{{'{\text{L}}}} } \right]$, where ${\varvec{\upbeta}}_{l}^{\text{S}}$ and ${\varvec{\upbeta}}_{l}^{\text{L}}$ summarize the effects of the Sarda and Lacaune gametes, respectively.

The model was tested at each SNP position by F-tests. Three null hypotheses were tested, ${\text{H}}_{0}$: ${\varvec{\upbeta}}_{l}$ = 0; ${\text{H}}_{0}$: ${\varvec{\upbeta}}_{l}^{\text{S}}$ = 0 and ${\text{H}}_{0}$: ${\varvec{\upbeta}}_{l}^{\text{L}}$ = 0. In the current study, only ${\text{BH}}^{\text{S}}$ tests, corresponding to ${\text{H}}_{0}$: ${\varvec{\upbeta}}_{l}^{\text{S}}$ = 0, will be analysed and discussed.

Genome-wide (GW) significance thresholds were determined by 2000 within-trait permutations of the residuals (${\varvec{\upvarepsilon}}$) of the reduce model ${\mathbf{y}} = \bf{1}\mu + {\mathbf{ZV}}_{g} {\varvec{\upalpha}} + {\varvec{\upvarepsilon}}$, where only the polygenic effects were considered. In order to break free from differences in the number of degrees of freedom at each SNP position, genome-wide maxima of the negative logarithms of p-values $[ - \log_{10} \left( {\text{p{-}value}} \right)]$ from each permutation were used to construct the null distribution.

## Results

### Production data and phenotypes for QTL detection

The ratios between genomic and total variance estimated by a single-trait animal model (Table 1) are consistent with the estimates of heritabilities for dairy traits in the literature [2]. Content traits were more heritable than yield traits. The most and the less heritable traits were PP and PY, respectively. APD and GEBV correlations between traits showed similar values and were consistent with phenotypic and genetic correlations reported in previous studies on sheep [2]. Strong correlations (from 0.88 to 0.95) were observed between yield traits, and moderate positive correlations were observed between content traits (0.58 and 0.62). MY was negatively correlated with both content traits, while the correlations of the other two yield traits with content traits were low.

**Table 1:** Ratios between genomic and total variance (diagonal, standard errors of estimates in brackets) and correlations between APD (above the diagonal) and between GEBV (below the diagonal)

Traits	MY	FY	PY	FP	PP
MY	0.35 (0.02)	0.90	0.95	− 0.32	− 0.34
FY	0.88	0.33 (0.02)	0.92	0.10	− 0.10
PY	0.93	0.91	0.31 (0.02)	− 0.15	− 0.03
FP	− 0.34	0.12	− 0.14	0.55 (0.02)	0.58
PP	− 0.42	− 0.14	− 0.08	0.62	0.61 (0.02)

*MY* milk yield, *FY* fat yield, *PY* protein yield, *FP* fat content, *PP* protein content

### Reconstruction of gametic phases

Preliminary editing of data led to remove SNPs with more than 5% missing genotypes and with a minor allele frequency (MAF) lower than 0.01. Only SNPs located on the 26 autosomes were retained.

The phasing procedure allowed the reconstruction of the sequence of the alleles carried by the investigated BH and RH for more than 99.5% of the SNP positions. After phasing, another 120 SNPs were excluded, because their genotypes were inconsistent with the phase estimated from neighbouring SNPs. Finally, 43,390 SNPs were retained for further analyses. The explored genomic portion was 2437 Mb long and the average distance between SNPs was 56 ± 49 kb with a maximum gap of 2.377 Mb on *Ovis aries* (OAR) chromosome 21.

### IBD probability calculation

On average, the maximum locus-wide ${\text{IBD}}_{\text{LA}}$ probability between each RH and all the BH was 0.99, which indicates the precise reconstruction of the meioses occurred across RP generations.

**Fig. 1:**
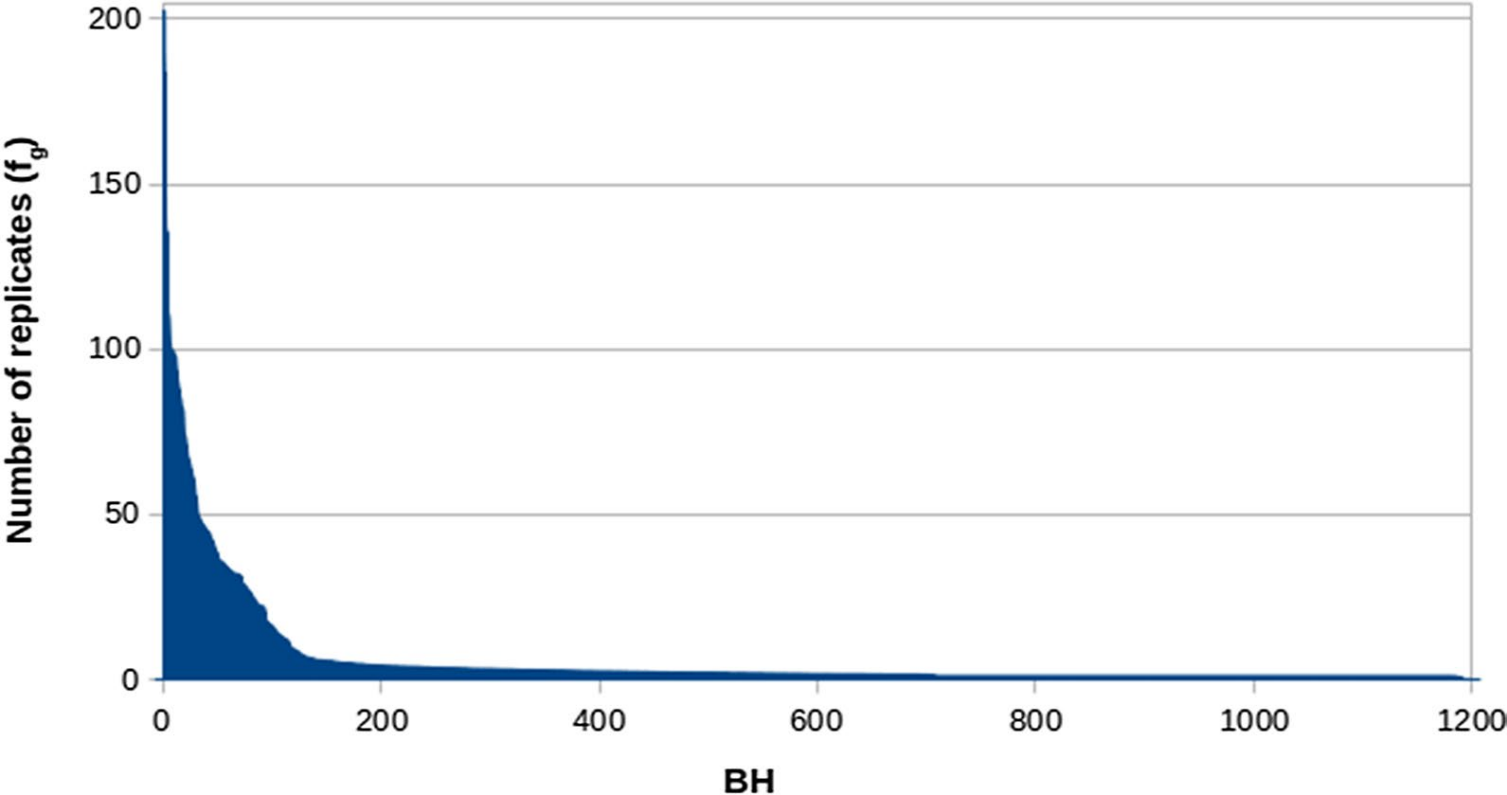
Genome-wide average number of replicates (${\mathbf{f}}_{g}$) per base gamete (BH, n = 1207) across the gametes of the resource population (RH; n = 7462)

**Fig. 2:**
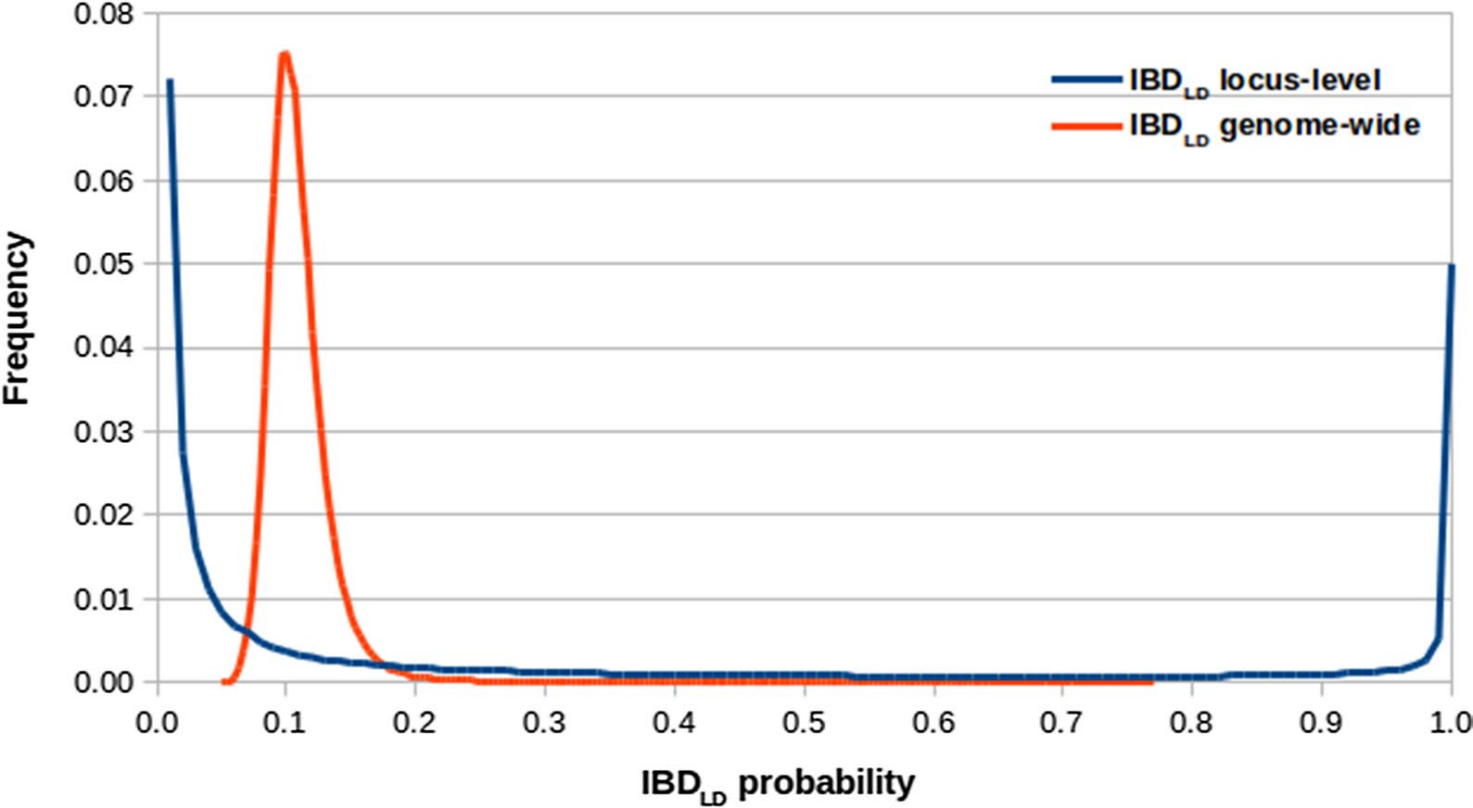
Distribution of non-zero ${\text{IBD}}_{\text{LD}}$ probabilities between base gametes (BH; n = 1207) at the locus (blue) and genome-wide (orange) levels

The distribution of the genome-wide number of replicates of the 1207 BH in RP (${\mathbf{f}}_{g}$) are depicted in Fig. 1. The impact of BH on RP was extremely variable, in fact the average number of replicates per BH ranged from 1 to 202. The locus-wide ${\text{IBD}}_{\text{LD}}$ probability between BH pairs was zero in 68% of cases. The distribution of non-zero ${\text{IBD}}_{\text{LD}}$ probabilities is shown in Fig. 2. The 13% and 6% of the locus-wide ${\text{IBD}}_{\text{LD}}$ were lower than 0.05 and higher than 0.95, respectively, which suggests that, for a large proportion of BH pairs, it would have been possible to approximate the IBD status to 0 or 1. However, another 13% of locus-wide ${\text{IBD}}_{\text{LD}}$ showed intermediate values, for which the approximation to 0 or 1 would have been less accurate.

Most of the BH pairs showed a genome-wide ${\text{IBD}}_{\text{LD}}$ probability equal to 0.10, with 95% having values ranging from 0.07 to 0.16 (Fig. 2). This result confirms that the original pool of Sarda gametes as well as the rams used to generate the yearly replacement of RP show a rather large genetic variability.

### Principal component analysis and QTL detection

**Fig. 3:**
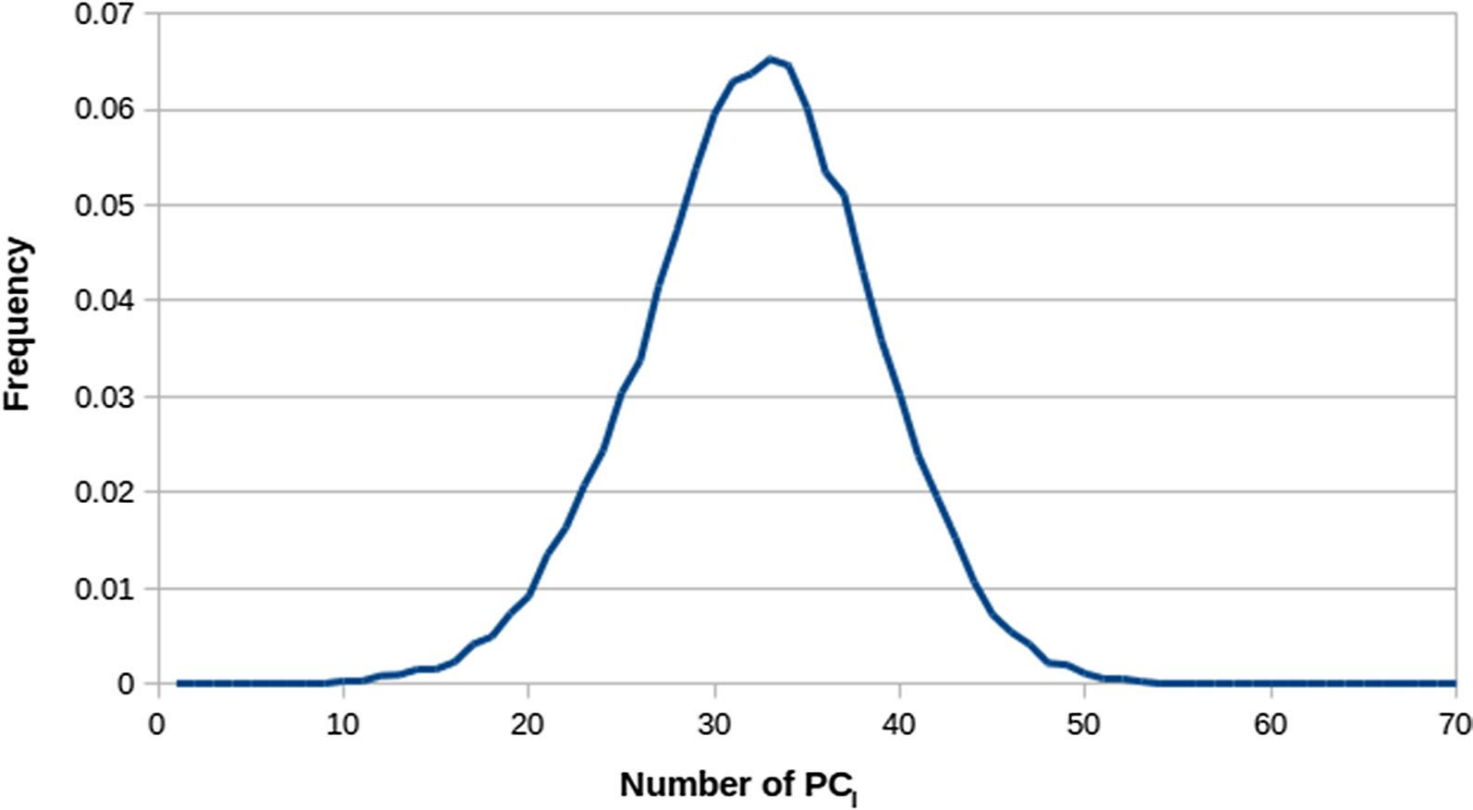
Frequencies across all loci (43,390 SNPs) of the number of principal components capturing 99% of the variation at the locus level (${\text{PC}}_{l}$)

The distribution of the number of PC needed to capture 99% of the locus-wide variability is shown in Fig. 3. The average number of ${\text{PC}}_{l}$ was 32.3 ± 6.4 with a maximum of 74 and a minimum of 9. As far as the breed of origin is concerned, the number of ${\text{PC}}_{l}$ explaining 99% of variation due to ${\text{BH}}^{\text{S}}$ (see Additional file 1) and ${\text{BH}}^{\text{L}}$ were 24.1 ± 6.0 and 8.2 ± 1.2, respectively.

Concerning the genome-wide analysis, 139 BH with the highest impact on RP (${\text{BH}}_{\text{h}}$) were selected on the basis of ${\mathbf{f}}_{g}$ and the genome-wide ${\text{IBD}}_{\text{LD}}$ probabilities between BH pairs. The ${\text{BH}}_{\text{h}}$ set included all 10 Lacaune gametes and 129 Sarda gametes. The sum of the original number of replicates (${\mathbf{f}}_{g}$) of ${\text{BH}}_{\text{h}}$ was 0.69 (i.e. 69% of the RH were replicates of ${\text{BH}}_{\text{h}}$). The remaining 30% of the RH variation was accounted for through the coefficients included in the ${\mathbf{W}}$ matrix and derived from ${\text{IBD}}_{\text{LD}}$ probabilities between ${\text{BH}}_{\text{h}}$ and other BH. At the genome-wide level, the number of ${\text{PC}}_{g}$ needed to capture 99% of the variation was 137.

The distributions of the genome-wide maxima of $- \log_{10} \left( {\text{p{-}value}} \right)$, corresponding to the null hypothesis ${\text{H}}_{0}$: ${\varvec{\upbeta}}_{l}^{\text{S}}$ = 0, obtained by 2000 within-trait permutations, did not show relevant differences across traits (see Additional file 2). The 5% threshold ranged from 5.59 to 5.69. Thus, the most conservative value, 5.69, was retained as the common GW threshold for all the analysed traits.

**Fig. 4:**
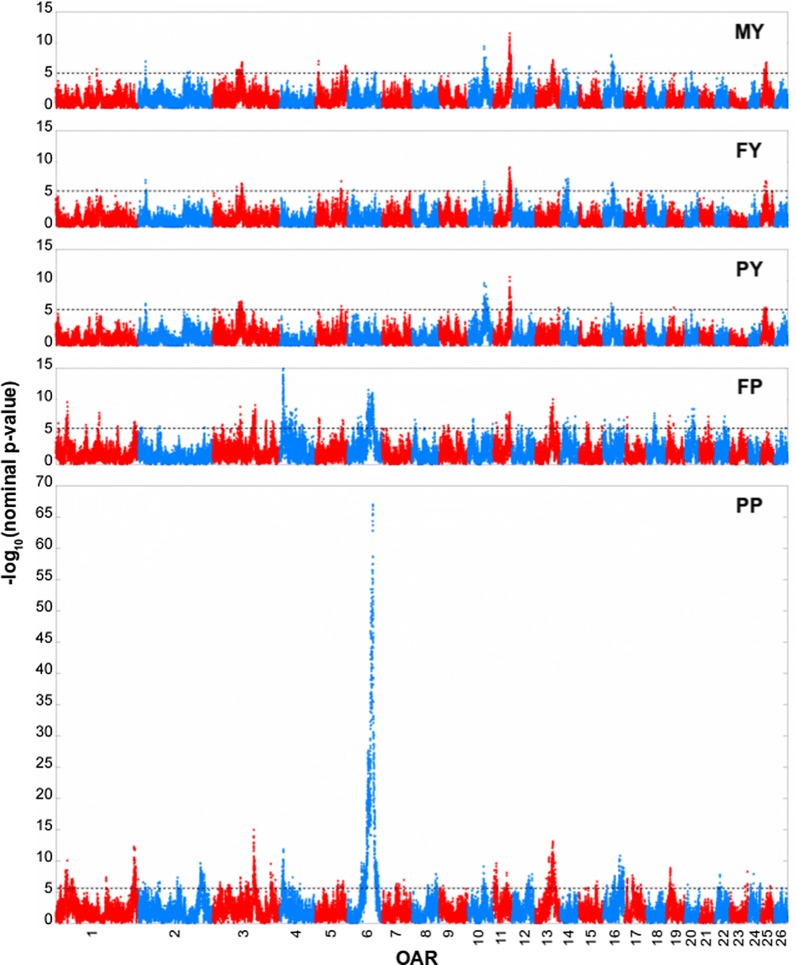
Manhattan plots showing − log10 (nominal p-values) corresponding to the null hypothesis that the effects of principal components that explain 99% of the variability due to the Sarda base gametes (BH^S^) at each locus (43,390 SNPs) are zero. The dashed black lines indicate the 0.05 genome-wide significance threshold determined by permutations. MY milk yield; FY fat yield; PY protein yield; FP fat content; PP protein content

Overall, 2563 positions exceeded the 0.05 GW significance threshold for at least one trait (Fig. 4) (see Additional file 3). There were 200, 108, 122, 918 and 1927 SNP positions significantly associated with MY, FY, PY, FP and PP, respectively. The number of significant positions affecting simultaneously one, two, three and four traits was 1943, 546, 56 and 18, respectively. Several significant positions were adjacent, which may be due to linkage disequilibrium between locations. In order to account for such dependency, significant positions were clustered into QTL regions (QTLR). The correlations between ${\mathbf{ZV}}_{l} {\varvec{\upbeta}}_{l}$ (corresponding to the second term of the model Eq. 1) were calculated for all pairs of significant SNPs on the same chromosome. Then, the strongest signal at the chromosome level was retained as the peak of the first QTLR. The peaks of further QTLR along the chromosome were iteratively identified among the significant locations that had correlations lower than 0.15 with the already defined QTLR peaks. Finally, the remaining significant positions were assigned to the QTLR with which they had the highest correlation. When QTLR for different traits overlapped, we considered them as a unique QTLR. This procedure may underestimate the true number of QTL if more than one gene affecting the trait(s) is located in the same genome region.

Details of the 75 defined QTLR are in Table 2. QTLR were detected across all 26 autosomes except OAR26. The largest number of QTLR (10) was detected on OAR1. Overall, 12, 11, 10, 46 and 43 QTLR significantly affected MY, FY, PY, FP and PP, respectively. Among these 75 QTLR, 46 were significant for one trait only: two for MY, two for FY, two for PY, 23 for FP and 17 for PP; 20 were significant for two traits: one for MY and FY, one for MY and PP and 18 for FP and PP; two QTLR were significant for three traits: one for MY and the two content traits and one for the three yield traits; five QTLR were significant for four traits: one for the three yield traits and FP, three for the three yield traits; and PP, and one for FY, PY, FP and PP; and finally two QTLR significant for all five investigated traits.

**Table 2:** Details of the QTL regions that include SNP positions exceeding the 0.05 genome-wide significance threshold

QTL region	OAR	Significant SNP (n)	Highest peak		Significant region (Mb)	Max − log10 (p-value)				
			SNP name	Pos. (Mb)		MY	FY	PY	FC	PC
1	1	1	rs422862154	8.20	8.2–8.2				6.4	
2	1	3	rs414745902	16.22	15.6–16.3					6.0
3	1	87	rs399459569	38.46	31.1–63.7				9.7	10.0
4	1	2	rs422745101	100.96	100.9–101.0				6.1	
5	1	1	rs402912954	136.40	136.4–136.4	6.0	5.8			
6	1	10	rs415285988	145.06	142.1–145.3				8.0	
7	1	4	rs424147980	168.06	168.1–168.1					7.4
8	1	2	rs430144352	206.80	206.4–206.8				5.9	
9	1	2	rs399774250	253.86	253.9–255.8					5.9
10	1	102	rs426289520	261.02	258.1–275.2				6.6	12.2
11	2	1	rs420740052	13.35	13.4–13.4				5.7	
12	2	2	rs420647200	16.66	16.7–22.7					6.5
13	2	5	rs406961044	24.31	24.0–27.2	7.2	7.3	6.5		5.7
14	2	1	rs420043297	33.17	33.2–33.2					5.7
15	2	5	rs428251930	71.40	65.4–71.8				5.9	6.6
16	2	8	rs404690479	131.48	130.1–140.8					7.3
17	2	134	rs403115176	207.86	204.9–223.2				5.8	9.6
18	3	1	rs400767835	4.26	4.3–4.3				5.8	
19	3	15	rs426591595	24.64	24.4–57.5				6.2	6.6
20	3	63	rs402979168	92.51	75.4–119.4	7.1	6.7	6.9	9.0	7.3
21	3	145	rs414469986	137.31	133.8–144.8				9.2	14.9
22	3	7	rs400309601	179.14	177.4–179.4				6.8	
23	3	39	rs425759731	194.13	193.8–209.9				6.7	9.5
24	4	131	rs421815167	12.34	5.9–24.9				14.9	11.8
25	4	52	rs426895887	55.13	30.6–55.3				8.6	7.3
26	4	7	rs414633478	68.14	66.6–78.1				6.1	6.3
27	5	2	rs419528574	10.52	10.5–10.5	7.3				
28	5	10	rs414853728	11.45	11.4–14.2				7.1	
29	5	27	rs404931334	86.91	72.6–93.1		7.1	6.1	6.9	6.8
30	5	10	rs405537538	101.53	100.1–106.9	6.6				
31	6	1	rs416743517	20.87	20.9–20.9				5.7	
32	6	1	rs406594979	22.39	22.4–22.4		5.7			
33	6	802	rs423823270	85.35	36.2–105.2				11.6	67.0
34	7	12	rs430671311	73.25	45.3–73.3					6.9
35	8	6	rs411259242	9.71	9.7–13.0				6.9	
36	8	24	rs404091172	79.80	52.9–84.6					7.9
37	9	1	rs423933809	13.53	13.5–13.5					6.0
38	9	1	rs422093338	14.86	14.9–14.9				5.9	
39	9	2	rs425782463	31.11	30.9–31.1				6.1	
40	10	7	rs427168327	19.79	18.3–20.2				7.1	
41	10	61	rs401955184	55.91	48.6–72.3	9.5	7.0	9.7		9.1
42	10	1	rs415670587	84.94	84.9–84.9				6.3	
43	11	48	rs428923302	9.85	0.7–14.2				6.5	9.6
44	11	104	rs425369179	55.43	43.1–60.3	11.6	9.2	10.7	8.1	8.1
45	12	1	rs423006875	13.91	13.9–13.9		5.9			
46	12	2	rs404434178	21.98	22.0–33.8				6.1	
47	12	29	rs404821945	68.04	51.3–68.1	6.5				7.8
48	13	223	rs406856069	58.58	36.5–73.8	7.4			10.1	13.1
49	13	2	rs418517103	78.12	78.1–78.1			5.9		
50	14	23	rs404164762	26.82	11.0–27.3	6.1	7.5	5.8	7.2	
51	14	7	rs425723410	50.19	33.9–50.3				7.4	
52	15	5	rs414954821	26.17	26.1–32.7				6.5	
53	15	3	rs421695633	61.28	56.4–61.3					6.7
54	15	2	rs421043654	76.61	76.6–76.6				5.9	
55	16	9	rs412271633	6.04	5.9–25.3				6.9	6.0
56	16	26	rs412025731	27.93	27.9–37.9	8.2	6.8	6.5		6.6
57	16	80	rs428170809	57.10	38.7–63.7				7.0	10.8
58	16	39	rs419400315	70.14	63.7–70.3				6.9	8.8
59	17	30	rs408602480	26.82	8.1–54.7				7.3	7.6
60	18	12	rs418457958	28.75	25.9–34.9				7.9	
61	19	27	rs425457738	13.15	6.7–15.3				7.5	8.8
62	19	1	rs406031789	22.79	22.8–22.8					6.1
63	19	3	rs399225729	23.72	23.7–23.8				6.3	
64	19	1	rs402952190	25.06	25.1–25.1			5.9		
65	20	28	rs422880779	25.70	4.4–35.3				8.6	6.2
66	21	1	rs400264754	20.17	20.2–20.2				5.7	
67	21	8	rs418090277	29.32	29.1–30.6				7.4	
68	22	1	rs427327212	10.86	10.9–10.9					5.7
69	22	7	rs161480899	18.07	18.0–24.2					7.7
70	22	6	rs399907821	33.75	31.1–38.9				7.4	
71	23	6	rs427932340	59.74	51.5–59.9					8.2
72	24	4	rs416153283	17.58	8.4–17.6					7.8
73	24	8	rs422576401	40.36	37.4–40.4					6.3
74	25	18	rs421872239	16.56	11.1–18.9	7.1	7.1	5.8		
75	25	1	rs405225833	31.48	31.5–31.5					6.0

QTL, *region* identifier; OAR, *Ovis aries* autosomes; *Significant SNP* (*n*) number of SNP positions exceeding the 0.05 genome-wide significance threshold [− log10(p-value) > 5.69] for at least for one trait; Highest peak, the most significant SNP across traits; SNP name, Pos. (Mb), name and position in Mb (from the ovine genome assembly v4.0 of the most significant SNP); Significant region (Mb), position in Mb of the first and last significant SNP of the QTL region; Max − log10 (p-value), highest significance per trait among the SNPs within a QTL region exceeding the genome-wide threshold of 0.05; − log10 (p-value), negative logarithm of the p-value corresponding to the null hypothesis that the effects of principal components that explain 99% of the variability due to the Sarda base gametes (BH^S^) are zero; MY, milk yield; FY, fat yield; PY, protein yield; FP, fat content; PP, protein content

The strongest signal was obtained for PP on OAR6 at 85.34 Mb where a nominal p-value of 11.12*10^−67^ was observed. The corresponding QTLR harboured significant positions also for FP and MY. The most significant position for FP (p-value = 1.26*10^−15^) was observed at 12.34 Mb on OAR4. This QTLR affected also PP. The most significant results for the three yield traits (p-value = 2.41*10^−12^, 5.89*10^−10^ and 2.20*10^−11^ for MY, FY and PY, respectively) were detected at 55.43 Mb on OAR11 where a significant peak for FP was also identified (Table 2).

## Discussion

Power, precision, robustness of QTL mapping experiments in complex populations may be affected by several issues (size of the experiment, number and frequency of base haplotypes, density of marker maps). As described above, the resource population investigated here is constituted by families based on male ancestors. LDLA mapping approaches are expected to be more suitable than linkage and genome-wide association analyses to fine map QTL regions in such populations. In fact, the LDLA analysis combines both the within-family linkage and population-wide linkage disequilibrium information to estimate IBD probabilities between haplotypes [51].

The proposed approach allowed us to solve the model by the least square method, which avoid a computational expensive variance component analysis. The advantages of approaches based on LDLA regression versus those on variance components, in term of ease of use and computing time, are well known [29] and have been clearly demonstrated by Roldand et al. [52].

In our study, LDLA mapping greatly benefits from the structure of the population, in which the ewes born after the first generation have both parents genotyped, which allows a precise reconstruction of the base gametes of the population and their transmission through generations. IBD information can be efficiently captured with PCA and the computational constraints due to the multi-collinearity generated by the high ${\text{IBD}}_{\text{LD}}$ probabilities that may occur between pairs of BH at the locus level can be overcome. The use of PCA avoided the implementation of prior clustering of BH or approximations in the IBD probability estimation [33, 34].

Moreover the strategy used here to collapse IBD information into principal components (i.e. the use of ${\mathbf{U}}_{l}^{w}$ instead of ${\mathbf{G}}_{l}$) is computationally efficient. It relies on the high precision of LA for defining the ancestral origin of each gamete, which is possible for populations with a strong familial structure. The effectiveness of the method in populations with weaker familial structure should be investigated.

Depending on the eigenvalues threshold used for PCs selection, the proposed approach allows to capture most of the IBD variation with a dramatic decrease in the number of effects to estimate. Although the direct solutions of the analysis are the effects of explanatory PC, the effect corresponding to each BH at position $l$ can be easily calculated (${\varvec{\upbeta}}_{l}^{\text{BH}} = {\mathbf{F}}_{l}^{ - 1/2} {\mathbf{V}}_{l}^{w} {\varvec{\upbeta}}_{l}$). Effects and frequencies of BH may be used as basic information to identify the BH that contribute most to the significance of a given locus. In fact, in their study on the identification of putative causative mutations that affect the protein content, Casu et al. [22] used such information to select individuals for whole-genome re-sequencing.

The QTL detection model proposed here included a fixed factor to adjust for the polygenic background of each individual based on the effect of PC that summarize the genome-wide BH variation. However, at the genome-wide level ${\text{IBD}}_{\text{LD}}$ probabilities between BH had moderate values when averaged across the genome. Indeed, most PC showed small eigenvalues and many of them were necessary to explain most of the variability. Thus, we applied an approach that aimed at reducing the number of BH to be included in the PCA to those that had the highest impact on RP (${\text{BH}}_{\text{h}}$) by taking their probability to be carried by an individual with a record into account.

During the development of our method, we applied it to the dataset that was simulated for the XVI QTLMAS meeting [53]. The results of QTL detection were very close to those reported by Garzia Gamez et al. [53] who used a more classical LDLA method [33, 53], which implemented variance component analysis and accounted for an individual random polygenic effect (see Additional file 4).

Overall, a large number of genomic regions that significantly affected milk traits were detected in this study. The number of detected regions largely exceeded those obtained by other LDLA studies in dairy sheep for milk production traits [16]. This larger number of detected QTLR is probably due to the larger size of the analyzed population. Indeed, Garzia-Gamez et al. [16] performed a LDLA mapping on a population of about 1700 Churra ewes that were organized in 16 half-sib families and they detected 34 genome-wide significant regions.

Consistent with the estimates of heritabilities, the number of QTLR that affected content traits was larger than that for yield traits. Several positions suggested pleiotropic effects. Twenty-nine QTLR affected more than one trait: nine affected at least two yield traits and frequently one or both of the content traits, four affected MY and both content traits, and 18 were significant for both content traits.

A very long list of positional candidate genes was obtained by overlapping the sheep genome reference (Oar_v4.0) with each QTLR. Overall, 745 annotated genes were detected but only a few of these were cited as dairy-related in previous studies on cattle [54] and sheep [55]. Among these, the most interesting genes were those in the casein cluster (*CSN1S1*, *CSN1S2*, *CSN2* and *CSN3*), which is mapped to OAR6 within the 85.00–85.23 Mb interval. This interval overlaps with the position of the strongest signal for PP found in this study. At almost the same position, a QTL for PP was detected in Churra sheep by GWA [21]. A deeper investigation of this region is ongoing by whole-genome re-sequencing of individuals that carry BH with large effects at the significant location. The aim is to list the candidate causative mutations by performing specific association studies of all the polymorphisms included in the genomic region [22].

The QTLR that affects PP and FP on OAR3 at 137.3 Mb overlaps with the *α*-*lactalbumin* gene (*LALBA*). This gene was previously reported as a strong candidate for PP and has been deeply investigated in the Churra breed [16, 21]. Two other interesting candidate genes are the *growth hormone receptor* (*GHR*) and *transcription factor AP*-*2 gamma* (*TFAP2C*) genes. *GHR* is located on OAR16 within the 31.83-32.00 Mb interval, where a QTLR that affects yield traits was detected. Previous studies in dairy cattle and sheep have shown that *GHR* affects milk production [54, 56]. *TFAP2C*, which is involved in the development, differentiation, and oncogenesis of the mammary gland [55], overlaps with a QTLR that is significant for MY, FP and PP on OAR13 at 58.6 Mb.

## Conclusions

We present a simple least square model to map QTL. It combines linkage disequilibrium and linkage analysis information and accounts for the polygenic effects of base gametes. The use of principal component analysis was found to be a good strategy to reduce the computational burden. A large number of regions associated to the variability of milk traits were identified. The outputs provided by this method are useful for the selection of individuals and genes that need to be further investigated for identifying causative mutations or markers in strong linkage disequilibrium with causative variants and for implementing them in genomic selection programs.

## Supplementary information

**Additional file 1.** Number of principal components (N.${\text{PC}}_{l}$) needed to explain more than 99% of the variability due to the Sarda base gametes (BH^S^) at each locus (43,390 SNPs). *OAR* (x-axis) *Ovis aries* autosomes.

**Additional file 2.** Distributions of the genome-wide maxima of −log_10_(p-values) obtained by 2000 within-trait permutations. −*Log*_*10*_*(p*-*values)* (x-axis) maximum across the genome (43,390 SNPs) of the negative logarithms of the p-values corresponding to the null hypothesis that the effects of principal components that explain 99% of the variability due to the Sarda base gametes (BH^S^) are zero; *MY* milk yield; *FY* fat yield; *PY* protein yield; *FP* fat content; *PP* protein content.

**Additional file 3.** Details on the SNP positions that exceed the genome-wide significance threshold of 0.05 for at least one trait. *QTL region* identifier; *OAR Ovis aries* autosomes; *SNP name* and *Position (bp)* name and position in base pairs of the significant SNP (from the ovine genome assembly v4.0); *n. of significant traits* number of traits for which the SNP exceeds the genome-wide threshold of 0.05 [−log_10_(p-value) > 5.69]; -*log*_*10*_*(p*-*value)* negative logarithm of the p-value corresponding to the null hypothesis that the effects of principal components that explain 99% of the variability due to the Sarda base gametes (BH^S^) are zero; *MY* milk yield; *FY* fat yield; *PY* protein yield; *FP* fat content; *PP* protein content.

**Additional file 4.** Application of the proposed method to the XVI QTLMAS simulated population data and comparison to LDLA results presented by Garcia-Gamez et al. [53]. Manhattan plots showing −log_10_(nominal p-values) corresponding to the null hypothesis that the effects of principal components that explain 99% of the variability due to base gametes of the XVI QTLMAS simulated population at each locus (10,000 SNPs) are zero. The dashed black lines indicate the 0.05 genome-wide significance threshold determined by Bonferroni correction for all the tests (10,000). Orange diamonds and grey vertical lines indicate the location of true simulated QTL [53]. Green triangles indicate QTL that were detected by variance component based LDLA mapping [33].

## Data Availability

The data that support the findings of this study are available from Centro Regionale di Programmazione (CRP), Regione Autonoma della Sardegna but restrictions apply to the availability of these data, which were used under license for the current study, and thus are not publicly available. However, data are available from the authors upon reasonable request and with permission of Centro Regionale di Programmazione (CRP), Regione Autonoma della Sardegna.

## Appendix

In this Appendix, we use a simple numerical example with few animals to explain how principal component analysis (PCA) is performed on IBD probability matrices. The example considers two males and 10 females with records and assumes that all the individuals are genotyped (Table 3).

**Table 3:** Pedigree and phenotypic records for the numerical example

id	Sire	Dam	Sex	Phenotype
1	–	–	M	–
2	–	–	M	–
3	1	–	F	0.180
4	2	–	F	0.796
5	2	3	F	0.972
6	1	4	F	0.631
7	2	3	F	0.823
8	1	4	F	0.796
9	1	5	F	0.545
10	2	6	F	0.972
11	2	7	F	0.068
12	1	8	F	0.807

**Table 4:** Classification of gametes

Gamete id^a^	Classification^b^
1p	BH
1m	BH
2p	BH
2m	BH
3p	RH
3m^c^	BH and RH
4p	RH
4m^c^	BH and RH
5p	RH
5m	RH
6p	RH
6m	RH
7p	RH
7m	RH
8p	RH
8m	RH
9p	RH
9m	RH
10p	RH
10m	RH
11p	RH
11m	RH
12p	RH
12m	RH

^a^Gamete ids are defined by the id of the individual with the subscript p or m, which denote the paternal or maternal origin, respectively

^b^Gametes are classified as base gametes (BH) when inherited by an ungenotyped parent or replicates of BH (RH) when inherited by a genotyped parent

^c^Gametes 3m and 4m are classified as BH since the dams of animals 3 and 4 were not genotyped; 3m and 4m will also be treated as RH (i.e. replicates of themselves) since they are associated to phenotypes

Thus, in this example there are 6 BH (${\text{n}}_{\text{BH}}$) and 20 RH (${\text{n}}_{\text{RH}}$, Table 4).

We assumed that the gametic phases of both BH and RH, for a map of $m$ markers, had been reconstructed by the linkage disequilibrium multilocus iterative peeling method [43] and that alleles assigned with a probability lower than 0.99 were set as missing.

### Analysis at the locus level

Let $l$ be one of the $m$ loci of the marker map. At this locus $l$, we assume that the grand-parental origin of each RH has been estimated with certainty by linkage analysis (LA), given the known gametic phases and the pedigree information (Table 5).

**Table 5:** Grand-parental origin of RH at locus $l$

RH	GS gamete	GD gamete	P(RH = GS)	P(RH = GD)
3p	1p	1m	1	0
3m		3m	0	1
4p	2p	2m	0	1
4m		4m	0	1
5p	2p	2m	1	0
5m	3p	3m	0	1
6p	1p	1m	0	1
6m	4p	4m	1	0
7p	2p	2m	1	0
7m	3p	3m	1	0
8p	1p	1m	0	1
8m	4p	4m	0	1
9p	1p	1m	1	0
9m	5p	5m	1	0
10p	2p	2m	0	1
10m	6p	6m	0	1
11p	2p	2m	1	0
11m	7p	7m	1	0
12p	1p	1m	0	1
12m	8p	8m	1	0

*GS* Grand-sire, *GD* Grand-dam, *P(RH = GS)* probability that RH is a replicate (i.e. identical-by-descent) of a GS gamete, *P(RH = GD)* probability that RH is a replicate (i.e. identical-by-descent) of a GD gamete

Thus, the ${\text{n}}_{\text{RH}} \times {\text{n}}_{\text{BH}}$ matrix of LA-based identity-by-descent (${\text{IBD}}_{\text{LA}}$) probabilities between RH and BH (${\mathbf{H}}_{l}$) calculated by the Fernando and Grossman procedure [46] from the grand-parental origin probabilities depicted in Table 5 is:

$${\mathbf{H}}_{l} = \left[ {\begin{array}{*{20}c} 1 & 0 & 0 & 0 & 0 & 0 \\ 0 & 0 & 0 & 0 & 1 & 0 \\ 0 & 0 & 0 & 1 & 0 & 0 \\ 0 & 0 & 0 & 0 & 0 & 1 \\ 0 & 0 & 1 & 0 & 0 & 0 \\ 0 & 0 & 0 & 0 & 1 & 0 \\ 0 & 1 & 0 & 0 & 0 & 0 \\ 0 & 0 & 0 & 1 & 0 & 0 \\ 0 & 0 & 1 & 0 & 0 & 0 \\ 1 & 0 & 0 & 0 & 0 & 0 \\ 0 & 1 & 0 & 0 & 0 & 0 \\ 0 & 0 & 0 & 0 & 0 & 1 \\ 1 & 0 & 0 & 0 & 0 & 0 \\ 0 & 0 & 1 & 0 & 0 & 0 \\ 0 & 0 & 0 & 1 & 0 & 0 \\ 0 & 0 & 0 & 1 & 0 & 0 \\ 0 & 0 & 1 & 0 & 0 & 0 \\ 0 & 0 & 1 & 0 & 0 & 0 \\ 0 & 1 & 0 & 0 & 0 & 0 \\ 0 & 1 & 0 & 0 & 0 & 0 \\ \end{array} } \right].$$

and the vector of ${\text{n}}_{\text{BH}}$ number of replicates (${\mathbf{f}}_{l}$) of each of BH across RH, calculated as ${\mathbf{f}}_{l} = {\mathbf{H}}_{l}^{'} 1$ (where $1$ is a vector of ${\text{n}}_{\text{RH}}$ ones), is:

$${\mathbf{f}}_{l} = \left[ {\begin{array}{*{20}c} 3 \\ 4 \\ 5 \\ 4 \\ 2 \\ 2 \\ \end{array} } \right].$$

Let us assume that the ${\text{n}}_{\text{BH}} \times {\text{n}}_{\text{BH}}$ matrix of LD-based identity-by-descent (${\text{IBD}}_{\text{LD}}$) probabilities between BH pairs, ${\mathbf{U}}_{l}$ is:

$${\mathbf{U}}_{l} = \left[ {\begin{array}{*{20}c} 1 & 0 & 0 & {0.15} & {0.9} & 0 \\ 0 & 1 & 0 & {0.75} & 0 & 1 \\ 0 & 0 & 1 & 0 & 0 & 0 \\ {0.15} & {0.75} & 0 & 1 & 0 & {0.75} \\ {0.9} & 0 & 0 & 0 & 1 & 0 \\ 0 & 1 & 0 & {0.75} & 0 & 1 \\ \end{array} } \right].$$

The ${\text{n}}_{\text{BH}} \times {\text{n}}_{\text{BH}}$ diagonal matrix ${\mathbf{F}}_{l}^{1/2}$ (in which the diagonal elements are the square root of the number of replicates stored in ${\mathbf{f}}_{l}$) is:

$${\mathbf{F}}_{l}^{1/2} = \left[ {\begin{array}{*{20}c} {1.73} & 0 & 0 & 0 & 0 & 0 \\ 0 & 2 & 0 & 0 & 0 & 0 \\ 0 & 0 & {2.24} & 0 & 0 & 0 \\ 0 & 0 & 0 & 2 & 0 & 0 \\ 0 & 0 & 0 & 0 & {1.41} & 0 \\ 0 & 0 & 0 & 0 & 0 & {1.41} \\ \end{array} } \right].$$

Then, the ${\text{n}}_{\text{BH}} \times {\text{n}}_{\text{BH}}$ ${\text{IBD}}_{\text{LD}}$ probabilities matrix weighted for the ${\text{IBD}}_{\text{LA}}$ probabilities (${\mathbf{U}}_{l}^{w}$), calculated as ${\mathbf{U}}_{l}^{w} = {\mathbf{F}}_{l}^{1/2} {\mathbf{U}}_{l} {\mathbf{F}}_{l}^{1/2}$ (Eq. (2)), is:

$${\mathbf{U}}_{l}^{w} = \left[ {\begin{array}{*{20}c} {3.00} & {0.00} & {0.00} & {0.52} & {2.20} & {0.00} \\ {0.00} & {4.00} & {0.00} & {3.00} & {0.00} & {2.83} \\ {0.00} & {0.00} & {5.00} & {0.00} & {0.00} & {0.00} \\ {0.52} & {3.00} & {0.00} & {4.00} & {0.00} & {2.12} \\ {2.20} & {0.00} & {0.00} & {0.00} & {2.00} & {0.00} \\ {0.00} & {2.83} & {0.00} & {2.12} & {0.00} & {2.00} \\ \end{array} } \right].$$

Eigenvalues of ${\mathbf{U}}_{l}^{w}$, defined by using the Jacobi algorithm, are in Table 6. The first four principal components capture more than 99% of the variability of ${\mathbf{U}}_{l}^{w}$ and, thus, are retained as explanatory (${\text{PC}}_{l}$).

**Table 6:** Eigenvalues of ${\mathbf{U}}_{l}^{w}$

Principal component	Eigenvalue	Variance explained (%)	Cumulative variance explained (%)
1	8.827	44.1	44.1
2	5.000	25.0	69.1
3	4.775	23.9	93.0
4	1.230	6.2	99.2
5	0.168	0.8	100.0
6	0.000	0.0	100.0

The ${\text{n}}_{\text{BH}} \times {\text{n}}_{{{\text{PC}}_{l} }}$ (6 × 4) matrix of eigenvectors extracted from ${\mathbf{U}}_{l}^{w}$ and relating BH with the four ${\text{PC}}_{l}$, ${\mathbf{V}}_{l}^{w}$ is:

$${\mathbf{V}}_{l}^{w} = \left[ {\begin{array}{*{20}c} {0.062} & {0.000} & {0.778} & { - 0.087} \\ {0.646} & {0.000} & { - 0.084} & {0.480} \\ {0.000} & {1.000} & {0.000} & {0.000} \\ {0.609} & {0.000} & {0.034} & { - 0.764} \\ {0.020} & {0.000} & {0.618} & {0.250} \\ {0.457} & {0.000} & { - 0.059} & {0.340} \\ \end{array} } \right].$$

Finally, the ${\text{n}}_{\text{RH}} \times {\text{n}}_{{{\text{PC}}_{l} }}$ (20 × 4) matrix (${\mathbf{V}}_{l}$) that allocates the ${\text{n}}_{{{\text{PC}}_{l} }}$ scores of RH, calculated as ${\mathbf{V}}_{l} = {\mathbf{H}}_{l} {\mathbf{F}}_{l}^{ - 1/2} {\mathbf{V}}_{l}^{w}$ (Eq. (3)), is thus:

$${\mathbf{V}}_{l} = \left[ {\begin{array}{*{20}c} {0.036} & {0.000} & {0.449} & { - 0.051} \\ {0.014} & {0.000} & {0.437} & {0.177} \\ {0.304} & {0.000} & {0.017} & { - 0.382} \\ {0.323} & {0.000} & { - 0.042} & {0.240} \\ {0.000} & {0.447} & {0.000} & {0.000} \\ {0.014} & {0.000} & {0.437} & {0.177} \\ {0.323} & {0.000} & { - 0.042} & {0.240} \\ {0.304} & {0.000} & {0.017} & { - 0.382} \\ {0.000} & {0.447} & {0.000} & {0.000} \\ {0.036} & {0.000} & {0.449} & { - 0.051} \\ {0.323} & {0.000} & { - 0.042} & {0.240} \\ {0.323} & {0.000} & { - 0.042} & {0.240} \\ {0.036} & {0.000} & {0.449} & { - 0.051} \\ {0.000} & {0.447} & {0.000} & {0.000} \\ {0.304} & {0.000} & {0.017} & { - 0.382} \\ {0.304} & {0.000} & {0.017} & { - 0.382} \\ {0.000} & {0.447} & {0.000} & {0.000} \\ {0.000} & {0.447} & {0.000} & {0.000} \\ {0.323} & {0.000} & { - 0.042} & {0.240} \\ {0.323} & {0.000} & { - 0.042} & {0.240} \\ \end{array} } \right].$$

### Principal component analysis on ${\text{G}}_{l}$

This section of the Appendix aims at demonstrating the equivalence between PCA that are carried out on ${\mathbf{U}}_{l}^{w}$ and PCA that are directly carried out on the LDLA IBD matrix ${\mathbf{G}}_{l}$ that was built as described by Meuwissen et al. [25].

A ${\text{n}}_{\text{RH}} \times {\text{n}}_{\text{RH}}$ ${\mathbf{G}}_{l}$ matrix can be calculated in our example by using ${\text{IBD}}_{\text{LD}}$ probabilities between BH pairs stored in ${\mathbf{U}}_{l}$ and grand-parental origin probabilities (Table 7). It allocates IBD probabilities between RH pairs obtained by combining LD and LA information. The ${\mathbf{G}}_{l}$ in our example is:

$${\mathbf{G}}_{l} = \left[\begin{array}{cccccccccccccccccccc}1&\;\;\;\;0.9&\;\;0.15&\;\;0&\;\;0&\;\;0.9&\;\;0&\;\;0.15&\;\;0&\;\;1&\;\;0&\;\;0&\;\;1&\;\;0&\;\;0.15&\;\;0.15&\;\;0&\;\;0&\;\;0&\;\;0\\ 0.9&\;\;1&\;\;0&\;\;0&\;\;0&\;\;1&\;\;0&\;\;0&\;\;0&\;\;0.9&\;\;0&\;\;0&\;\;0.9&\;\;0&\;\;0&\;\;0&\;\;0&\;\;0&\;\;0&\;\;0\\ 0.15&\;\;0&\;\;1&\;\;0.75&\;\;0&\;\;0&\;\;0.75&\;\;1&\;\;0&\;\;0.15&\;\;0.75&\;\;0.75&\;\;0.15&\;\;0&\;\;1&\;\;1&\;\;0&\;\;0&\;\;0.75&\;\;0.75\\ 0&\;\;0&\;\;0.75&\;\;1&\;\;0&\;\;0&\;\;1&\;\;0.75&\;\;0&\;\;0&\;\;1&\;\;1&\;\;0&\;\;0&\;\;0.75&\;\;0.75&\;\;0&\;\;0&\;\;1&\;\;1\\ 0&\;\;0&\;\;0&\;\;0&\;\;1&\;\;0&\;\;0&\;\;0&\;\;1&\;\;0&\;\;0&\;\;0&\;\;0&\;\;1&\;\;0&\;\;0&\;\;1&\;\;1&\;\;0&\;\;0\\ 0.9&\;\;1&\;\;0&\;\;0&\;\;0&\;\;1&\;\;0&\;\;0&\;\;0&\;\;0.9&\;\;0&\;\;0&\;\;0.9&\;\;0&\;\;0&\;\;0&\;\;0&\;\;0&\;\;0&\;\;0\\ 0&\;\;0&\;\;0.75&\;\;1&\;\;0&\;\;0&\;\;1&\;\;0.75&\;\;0&\;\;0&\;\;1&\;\;1&\;\;0&\;\;0&\;\;0.75&\;\;0.75&\;\;0&\;\;0&\;\;1&\;\;1\\ 0.15&\;\;0&\;\;1&\;\;0.75&\;\;0&\;\;0&\;\;0.75&\;\;1&\;\;0&\;\;0.15&\;\;0.75&\;\;0.75&\;\;0.15&\;\;0&\;\;1&\;\;1&\;\;0&\;\;0&\;\;0.75&\;\;0.75\\ 0&\;\;0&\;\;0&\;\;0&\;\;1&\;\;0&\;\;0&\;\;0&\;\;1&\;\;0&\;\;0&\;\;0&\;\;0&\;\;1&\;\;0&\;\;0&\;\;1&\;\;1&\;\;0&\;\;0\\ 1&\;\;0.9&\;\;0.15&\;\;0&\;\;0&\;\;0.9&\;\;0&\;\;0.15&\;\;0&\;\;1&\;\;0&\;\;0&\;\;1&\;\;0&\;\;0.15&\;\;0.15&\;\;0&\;\;0&\;\;0&\;\;0\\ 0&\;\;0&\;\;0.75&\;\;1&\;\;0&\;\;0&\;\;1&\;\;0.75&\;\;0&\;\;0&\;\;1&\;\;1&\;\;0&\;\;0&\;\;0.75&\;\;0.75&\;\;0&\;\;0&\;\;1&\;\;1\\ 0&\;\;0&\;\;0.75&\;\;1&\;\;0&\;\;0&\;\;1&\;\;0.75&\;\;0&\;\;0&\;\;1&\;\;1&\;\;0&\;\;0&\;\;0.75&\;\;0.75&\;\;0&\;\;0&\;\;1&\;\;1\\ 1&\;\;0.9&\;\;0.15&\;\;0&\;\;0&\;\;0.9&\;\;0&\;\;0.15&\;\;0&\;\;1&\;\;0&\;\;0&\;\;1&\;\;0&\;\;0.15&\;\;0.15&\;\;0&\;\;0&\;\;0&\;\;0\\ 0&\;\;0&\;\;0&\;\;0&\;\;1&\;\;0&\;\;0&\;\;0&\;\;1&\;\;0&\;\;0&\;\;0&\;\;0&\;\;1&\;\;0&\;\;0&\;\;1&\;\;1&\;\;0&\;\;0\\ 0.15&\;\;0&\;\;1&\;\;0.75&\;\;0&\;\;0&\;\;0.75&\;\;1&\;\;0&\;\;0.15&\;\;0.75&\;\;0.75&\;\;0.15&\;\;0&\;\;1&\;\;1&\;\;0&\;\;0&\;\;0.75&\;\;0.75\\ 0.15&\;\;0&\;\;1&\;\;0.75&\;\;0&\;\;0&\;\;0.75&\;\;1&\;\;0&\;\;0.15&\;\;0.75&\;\;0.75&\;\;0.15&\;\;0&\;\;1&\;\;1&\;\;0&\;\;0&\;\;0.75&\;\;0.75\\ 0&\;\;0&\;\;0&\;\;0&\;\;1&\;\;0&\;\;0&\;\;0&\;\;1&\;\;0&\;\;0&\;\;0&\;\;0&\;\;1&\;\;0&\;\;0&\;\;1&\;\;1&\;\;0&\;\;0\\ 0&\;\;0&\;\;0&\;\;0&\;\;1&\;\;0&\;\;0&\;\;0&\;\;1&\;\;0&\;\;0&\;\;0&\;\;0&\;\;1&\;\;0&\;\;0&\;\;1&\;\;1&\;\;0&\;\;0\\ 0&\;\;0&\;\;0.75&\;\;1&\;\;0&\;\;0&\;\;1&\;\;0.75&\;\;0&\;\;0&\;\;1&\;\;1&\;\;0&\;\;0&\;\;0.75&\;\;0.75&\;\;0&\;\;0&\;\;1&\;\;1\\ 0&\;\;0&\;\;0.75&\;\;1&\;\;0&\;\;0&\;\;1&\;\;0.75&\;\;0&\;\;0&\;\;1&\;\;1&\;\;0&\;\;0&\;\;0.75&\;\;0.75&\;\;0&\;\;0&\;\;1&\;\;1\end{array}\right].$$

**Table 7:** Eigenvalues of ${\mathbf{G}}_{l}$

Principal component	Eigenvalue	Variance explained (%)	Cumulative variance explained (%)
1	8.827	44.1	44.1
2	5.000	25.0	69.1
3	4.775	23.9	93.0
4	1.230	6.2	99.2
5	0.168	0.8	100.0
6	0.000	0.0	100.0
7	0.000	0.0	100.0
8	0.000	0.0	100.0
9	0.000	0.0	100.0
10	0.000	0.0	100.0
11	0.000	0.0	100.0
12	0.000	0.0	100.0
13	0.000	0.0	100.0
14	0.000	0.0	100.0
15	0.000	0.0	100.0
16	0.000	0.0	100.0
17	0.000	0.0	100.0
18	0.000	0.0	100.0
19	0.000	0.0	100.0
20	0.000	0.0	100.0

Eigenvalues of ${\mathbf{G}}_{l}$, calculated by using the Jacobi algorithm, are depicted in Table 7. The eigenvalues of the first five PC (i.e. those not equal to zero) overlap precisely with those obtained with ${\mathbf{U}}_{l}^{w}$ (Table 6). Thus, even in this case the first four PC are retained as explanatory (${\text{PC}}_{l}$).

The ${\text{PC}}_{l}$ score of RH extracted from ${\mathbf{G}}_{l}$ and stored in a ${\text{n}}_{\text{RH}} \times {\text{n}}_{{{\text{PC}}_{l} }}$ matrix (say ${\mathbf{V}}_{l}^{G}$) are:

$${\mathbf{V}}_{l}^{G} = \left[ {\begin{array}{*{20}c} {0.036} & {0.000} & {0.449} & { - 0.051} \\ {0.014} & {0.000} & {0.437} & {0.177} \\ {0.304} & {0.000} & {0.017} & { - 0.382} \\ {0.323} & {0.000} & { - 0.042} & {0.240} \\ {0.000} & {0.447} & {0.000} & {0.000} \\ {0.014} & {0.000} & {0.437} & {0.177} \\ {0.323} & {0.000} & { - 0.042} & {0.240} \\ {0.304} & {0.000} & {0.017} & { - 0.382} \\ {0.000} & {0.447} & {0.000} & {0.000} \\ {0.036} & {0.000} & {0.449} & { - 0.051} \\ {0.323} & {0.000} & { - 0.042} & {0.240} \\ {0.323} & {0.000} & { - 0.042} & {0.240} \\ {0.036} & {0.000} & {0.449} & { - 0.051} \\ {0.000} & {0.447} & {0.000} & {0.000} \\ {0.304} & {0.000} & {0.017} & { - 0.382} \\ {0.304} & {0.000} & {0.017} & { - 0.382} \\ {0.000} & {0.447} & {0.000} & {0.000} \\ {0.000} & {0.447} & {0.000} & {0.000} \\ {0.323} & {0.000} & { - 0.042} & {0.240} \\ {0.323} & {0.000} & { - 0.042} & {0.240} \\ \end{array} .} \right]$$

Even in this case, ${\mathbf{V}}_{l}^{G}$ precisely overlap ${\mathbf{V}}_{l}$. demonstrating that in terms of results the PCA carried out on ${\mathbf{U}}_{l}^{w}$ is equivalent to the PCA carried out directly on ${\mathbf{G}}_{l}$. Nevertheless, PCA on ${\mathbf{U}}_{l}^{w}$ is much faster and less computationally demanding since it refers to a much smaller matrix ${\mathbf{G}}_{l}$.

### Analysis at the genome-wide level

The ${\text{n}}_{\text{RH}} \times {\text{n}}_{\text{BH}}$ genome-wide ${\text{IBD}}_{\text{LA}}$ probabilities matrix ${\mathbf{H}}_{g}$, should be calculated, in a map with $m$ markers, as ${\mathbf{H}}_{g} = \left( {{\mathbf{H}}_{1} + {\mathbf{H}}_{2} + {\mathbf{H}}_{3} + \cdots + {\mathbf{H}}_{m} } \right)/m$. In this example, we assume that ${\mathbf{H}}_{g}$ has the following final configuration:

$${\mathbf{H}}_{g} = \left[ {\begin{array}{*{20}c} {0.5} & {0.5} & 0 & 0 & 0 & 0 \\ 0 & 0 & 0 & 0 & 1 & 0 \\ 0 & 0 & {0.5} & {0.5} & 0 & 0 \\ 0 & 0 & 0 & 0 & 0 & 1 \\ 0 & 0 & {0.5} & {0.5} & 0 & 0 \\ {0.25} & {0.25} & 0 & 0 & {0.5} & 0 \\ {0.5} & {0.5} & 0 & 0 & 0 & 0 \\ 0 & 0 & {0.25} & {0.25} & 0 & {0.5} \\ 0 & 0 & {0.5} & {0.5} & 0 & 0 \\ {0.25} & {0.25} & 0 & 0 & {0.5} & 0 \\ {0.5} & {0.5} & 0 & 0 & 0 & 0 \\ 0 & 0 & {0.25} & {0.25} & 0 & {0.5} \\ {0.5} & {0.5} & 0 & 0 & 0 & 0 \\ {0.125} & {0.125} & {0.25} & {0.25} & {0.25} & 0 \\ 0 & 0 & {0.5} & {0.5} & 0 & 0 \\ {0.25} & {0.25} & {0.125} & {0.125} & 0 & {0.25} \\ 0 & 0 & {0.5} & {0.5} & 0 & 0 \\ {0.125} & {0.125} & {0.25} & {0.25} & {0.25} & 0 \\ {0.5} & {0.5} & 0 & 0 & 0 & 0 \\ {0.25} & {0.25} & {0.125} & {0.125} & 0 & {0.25} \\ \end{array} } \right],$$

and the vector of ${\text{n}}_{\text{BH}}$ genome-wide average number of replicates (${\mathbf{f}}_{g}$) of BH over RH (i.e. the average number of replicates of each BH across-genome), which should be calculated as ${\mathbf{f}}_{g} = \left( {{\mathbf{f}}_{1} + {\mathbf{f}}_{2} + {\mathbf{f}}_{3} + \cdots + {\mathbf{f}}_{m} } \right)/m$ or, more simply, as ${\mathbf{f}}_{g} = {\mathbf{H}}_{g}^{\varvec{'}}$ **1** (where $1$ is a vector of ${\text{n}}_{\text{RH}}$ ones), is:

$${\mathbf{f}}_{g} = \left[ {\begin{array}{*{20}c} {3.75} \\ {3.75} \\ {3.75} \\ {3.75} \\ {2.5} \\ {2.5} \\ \end{array} } \right].$$

The ${\text{n}}_{\text{BH}} \times {\text{n}}_{\text{BH}}$ genome-wide ${\text{IBD}}_{\text{LD}}$ probabilities ${\mathbf{U}}_{g}$, which in real data is calculated as ${\mathbf{U}}_{g} = \left( {{\mathbf{U}}_{1} + {\mathbf{U}}_{2} + {\mathbf{U}}_{3} + \cdots + {\mathbf{U}}_{m} } \right)/m$, is assumed here to have the following final configuration:

$${\mathbf{U}}_{g} = \left[ {\begin{array}{*{20}c} 1 & {0.05} & 0 & {0.1} & {0.25} & {0.1} \\ {0.05} & 1 & 0 & {0.1} & {0.1} & {0.15} \\ 0 & 0 & 1 & 0 & 0 & 0 \\ {0.1} & {0.1} & 0 & 1 & {0.1} & {0.1} \\ {0.25} & {0.1} & 0 & {0.1} & 1 & {0.1} \\ {0.1} & {0.15} & 0 & {0.1} & {0.1} & 1 \\ \end{array} } \right].$$

Note that, we assumed that genome-wide (and thus all the locus-wide) ${\text{IBD}}_{\text{LD}}$ probabilities between the BH 2p and all other BH are zero (third row and third column) in order to mimic a gamete from another breed. Moreover, the off-diagonal elements in ${\mathbf{U}}_{g}$ have more moderate values than those which may arise in ${\mathbf{U}}_{l}$. Because of this, PCA performed on a matrix ${\mathbf{U}}_{g}^{w}$ (calculated as described above for ${\mathbf{U}}_{l}^{w}$) does not result in a dimensional reduction as it was observed at the locus level (Table 6).

Let us assume that 1p, 1m, 2p, 2m have been selected as the base gametes with the highest impact (${\text{BH}}_{\text{h}}$) on the population (the demonstration of how ${\text{BH}}_{\text{h}}$ are selected in the real data would require a population much larger than that simulated here). The ${\text{n}}_{\text{BH}} \times {\text{n}}_{{{\text{BH}}_{\text{h}} }}$ section of ${\mathbf{U}}_{g}$ corresponding to the genome-wide ${\text{IBD}}_{\text{LD}}$ probabilities between all the BH with ${\text{BH}}_{\text{h}}$, and denoted as ${\mathbf{U}}_{g\_h}$, is:

$${\mathbf{U}}_{g\_h} = \left[ {\begin{array}{*{20}c} 1 & {0.05} & 0 & {0.1} \\ {0.05} & 1 & 0 & {0.1} \\ 0 & 0 & 1 & 0 \\ {0.1} & {0.1} & 0 & 1 \\ {0.25} & {0.1} & 0 & {0.1} \\ {0.1} & {0.15} & 0 & {0.1} \\ \end{array} } \right],$$

and the ${\text{n}}_{{{\text{BH}}_{\text{h}} }} \times {\text{n}}_{{{\text{BH}}_{\text{h}} }}$ matrix ${\mathbf{U}}_{g\_hh}$ corresponding to the portion of ${\mathbf{U}}_{g}$. including ${\text{IBD}}_{\text{LD}}$. probabilities between ${\text{BH}}_{\text{h}}$ pairs is:

$${\mathbf{U}}_{g\_hh} = \left[ {\begin{array}{*{20}c} 1 & {0.05} & 0 & {0.1} \\ {0.05} & 1 & 0 & {0.1} \\ 0 & 0 & 1 & 0 \\ {0.1} & {0.1} & 0 & 1 \\ \end{array} } \right].$$

The ${\text{n}}_{\text{BH}} \times {\text{n}}_{{{\text{BH}}_{\text{h}} }}$ matrix ${\mathbf{W}}$, including coefficients relating ${\text{BH}}_{\text{h}}$ to all the BH and calculated as: ${\mathbf{W}} = {\mathbf{U}}_{g\_h} {\mathbf{U}}_{g\_hh}^{ - 1}$ (Eq (4)), is thus:

$${\mathbf{W}} = \left[ {\begin{array}{*{20}c} {1.00} & {0.00} & {0.00} & {0.00} \\ {0.00} & {1.00} & {0.00} & {0.00} \\ {0.00} & {0.00} & {1.00} & {0.00} \\ {0.00} & {0.00} & {0.00} & {1.00} \\ {0.24} & {0.08} & {0.00} & {0.07} \\ {0.09} & {0.14} & {0.00} & {0.08} \\ \end{array} } \right].$$

The vector of ${\text{n}}_{{{\text{BH}}_{\text{h}} }}$ updated number of replicates of ${\text{BH}}_{\text{h}}$ (${\mathbf{f}}_{g\_h}$) calculated as ${\mathbf{f}}_{g\_h} = {\mathbf{W^{\prime}f}}_{g}$ is:

$${\mathbf{f}}_{g\_h} = \left[ {\begin{array}{*{20}c} {4.56} \\ {4.30} \\ {3.75} \\ {4.11} \\ \end{array} } \right].$$

Note that $\sum {\mathbf{f}}_{g\_h} /{\text{n}}_{\text{RH}}$ = 16.723/20 = 0.83 (this is the best value that can be obtained in this simulation but it is far away from the 0.99 obtained with real data).

The ${\text{n}}_{{{\text{BH}}_{\text{h}} }} \times {\text{n}}_{{{\text{BH}}_{\text{h}} }}$ diagonal matrix ${\mathbf{F}}_{g\_h}^{1/2}$ (in which the diagonal elements are the root square of the average number of replicates stored in ${\mathbf{f}}_{g\_h}$) is:

$${\mathbf{F}}_{g\_h}^{1/2} = \left[ {\begin{array}{*{20}c} {2.136} & 0 & 0 & 0 \\ 0 & {2.073} & 0 & 0 \\ 0 & 0 & {1.936} & 0 \\ 0 & 0 & 0 & {2.028} \\ \end{array} } \right].$$

Finally, the ${\text{n}}_{{{\text{BH}}_{\text{h}} }} \times {\text{n}}_{{{\text{BH}}_{\text{h}} }}$ matrix ${\mathbf{U}}_{g\_hh}^{w}$ calculated as ${\mathbf{U}}_{g\_hh}^{w} = {\mathbf{F}}_{g\_h}^{1/2} {\mathbf{U}}_{g\_hh} {\mathbf{F}}_{g\_h}^{1/2}$ (Eq. (5)), is:

$${\mathbf{U}}_{g\_hh}^{\text{w}} = \left[ {\begin{array}{*{20}c} {4.561} & {0.221} & {0.000} & {0.433} \\ {0.221} & {4.298} & {0.000} & {0.421} \\ {0.000} & {0.000} & {3.750} & {0.000} \\ {0.433} & {0.421} & {0.000} & {4.114} \\ \end{array} } \right].$$

Eigenvalues of ${\mathbf{U}}_{g\_hh}^{w}$, which are calculated by using the Jacobi algorithm, are in Table 8. In this example, all four principal components are needed to capture more than 99% of the variability of ${\mathbf{U}}_{g\_hh}^{w}$ and thus are retained as explanatory (${\text{PC}}_{g}$).

**Table 8:** Eigenvalues of ${\mathbf{U}}_{g\_hh}^{\text{w}}$

Principal component	Eigenvalue	Variance explained (%)	Cumulative variance explained (%)
1	5.059	30.3	30.3
2	4.201	25.1	55.4
3	3.750	22.4	77.8
4	3.713	22.2	100.0

The ${\text{n}}_{{{\text{BH}}_{\text{h}} }} \times {\text{n}}_{{{\text{PC}}_{g} }}$ (4 × 4) matrix of eigenvectors extracted from ${\mathbf{U}}_{g\_hh}^{w}$, and relating ${\text{BH}}_{\text{h}}$ with the four ${\text{PC}}_{g}$, ${\mathbf{V}}_{g\_hh}^{w}$ is:

$${\mathbf{V}}_{g\_hh}^{w} = \left[ {\begin{array}{*{20}c} {0.685} & { - 0.666} & {0.000} & { - 0.296} \\ {0.495} & {0.723} & {0.000} & { - 482} \\ {0.000} & {0.000} & {1.000} & {0.000} \\ {0.535} & {0.184} & {0.000} & {0.825} \\ \end{array} } \right].$$

Finally, the ${\text{n}}_{{{\text{RH}}_{\text{h}} }} \times {\text{n}}_{{{\text{PC}}_{g} }}$ (20 × 4) matrix (${\mathbf{V}}_{g}$) allocating the ${\text{PC}}_{l}$ scores of RH, calculated as ${\mathbf{V}}_{g} = {\mathbf{H}}_{g} {\mathbf{WF}}_{g\_h}^{ - 1/2} {\mathbf{V}}_{g\_hh}^{w}$ (Eq. (6)), is thus:

$${\mathbf{V}}_{g} = \varvec{ }\left[ {\begin{array}{*{20}c} {0.280} & {0.018} & {0.000} & { - 0.186} \\ {0.114} & { - 0.040} & {0.000} & { - 0.024} \\ {0.132} & {0.045} & {0.258} & {0.203} \\ {0.081} & {0.029} & {0.000} & { - 0.012} \\ {0.132} & {0.045} & {0.258} & {0.203} \\ {0.197} & { - 0.011} & {0.000} & { - 0.105} \\ {0.280} & {0.018} & {0.000} & { - 0.186} \\ {0.106} & {0.037} & {0.129} & {0.096} \\ {0.132} & {0.045} & {0.258} & {0.203} \\ {0.197} & { - 0.011} & {0.000} & { - 0.105} \\ {0.280} & {0.018} & {0.000} & { - 0.186} \\ {0.106} & {0.037} & {0.129} & {0.096} \\ {0.280} & {0.018} & {0.000} & { - 0.186} \\ {0.164} & {0.017} & {0.129} & {0.049} \\ {0.132} & {0.045} & {0.258} & {0.203} \\ {0.193} & {0.028} & {0.065} & { - 0.045} \\ {0.132} & {0.045} & {0.258} & {0.203} \\ {0.164} & {0.017} & {0.129} & {0.049} \\ {0.280} & {0.018} & {0.000} & { - 0.186} \\ {0.193} & {0.028} & {0.065} & { - 0.045} \\ \end{array} } \right].$$
