# Supplementary figures and images for: Mapping genomic regions affecting milk traits in Sarda sheep by using the OvineSNP50 Beadchip and principal components to perform combined linkage and linkage disequilibrium analysis
Source: Genet Sel Evol. 2019 Nov 19;51:65. doi: 10.1186/s12711-019-0508-0 (PMC6862840; doi:10.1186/s12711-019-0508-0)

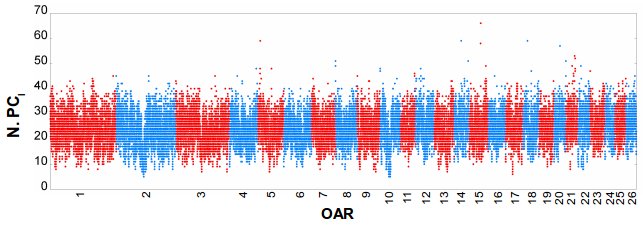

Supplement: Supplementary file 1 — Additional file 1. Number of principal components (N.${\text{PC}}_{l}$PCl) needed to explain more than 99% of the variability due to the Sarda base gametes (BHS) at each locus (43,390 SNPs). OAR (x-axis) Ovis aries autosomes. [file 12711_2019_508_MOESM1_ESM.png]

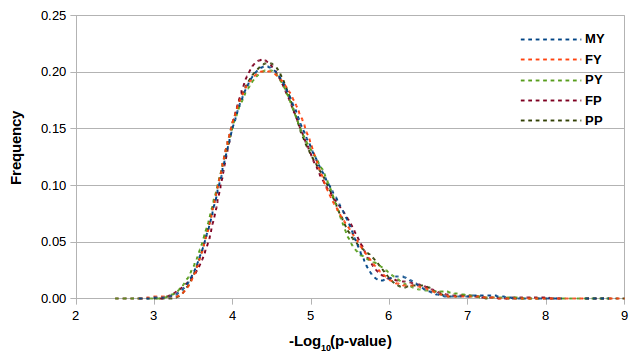

Supplement: Supplementary file 2 — Additional file 2. Distributions of the genome-wide maxima of −log10(p-values) obtained by 2000 within-trait permutations. −Log10(p-values) (x-axis) maximum across the genome (43,390 SNPs) of the negative logarithms of the p-values corresponding to the null hypothesis that the effects of principal components that explain 99% of the variability due to the Sarda base gametes (BHS) are zero; MY milk yield; FY fat yield; PY protein yield; FP fat content; PP protein content. [file 12711_2019_508_MOESM2_ESM.png]

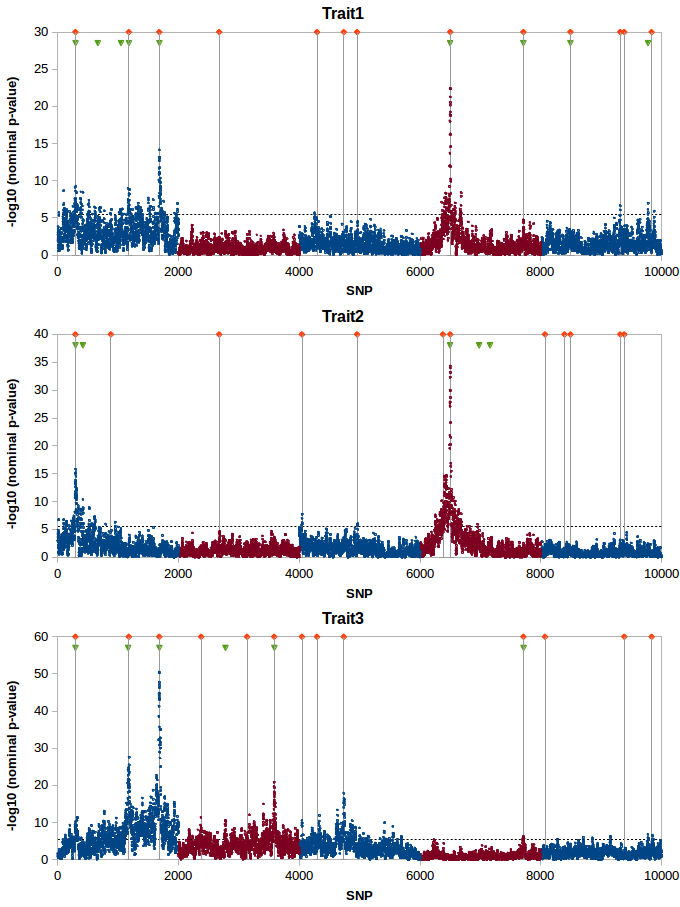

Supplement: Supplementary file 4 — Additional file 4. Application of the proposed method to the XVI QTLMAS simulated population data and comparison to LDLA results presented by Garcia-Gamez et al. [53]. Manhattan plots showing −log10(nominal p-values) corresponding to the null hypothesis that the effects of principal components that explain 99% of the variability due to base gametes of the XVI QTLMAS simulated population at each locus (10,000 SNPs) are zero. The dashed black lines indicate the 0.05 genome-wide significance threshold determined by Bonferroni correction for all the tests (10,000). Orange diamonds and grey vertical lines indicate the location of true simulated QTL [53]. Green triangles indicate QTL that were detected by variance component based LDLA mapping [33]. [file 12711_2019_508_MOESM4_ESM.png]
